# Autonomous Visualization of Damage in Polymers by Metal‐Free Polymerizations of Microencapsulated Activated Alkynes

**DOI:** 10.1002/advs.202105395

**Published:** 2022-01-23

**Authors:** Ting Han, Shusheng Chen, Xinnan Wang, Xinyao Fu, Haifei Wen, Zaiyu Wang, Dong Wang, Anjun Qin, Jinglei Yang, Ben Zhong Tang

**Affiliations:** ^1^ Center for AIE Research Shenzhen Key Laboratory of Polymer Science and Technology Guangdong Research Center for Interfacial Engineering of Functional Materials College of Materials Science and Engineering Shenzhen University Shenzhen 518060 China; ^2^ Department of Mechanical and Aerospace Engineering The Hong Kong University of Science and Technology Clear Water Bay Kowloon Hong Kong 999077 China; ^3^ Department of Chemistry Hong Kong Branch of Chinese National Engineering Research Center for Tissue Restoration and Reconstruction The Hong Kong University of Science and Technology Clear Water Bay Kowloon Hong Kong 999077 China; ^4^ State Key Laboratory of Luminescent Materials and Devices Guangdong Provincial Key Laboratory of Luminescence from Molecular Aggregates SCUT‐HKUST Joint Research Institute AIE Institute Center for Aggregation‐Induced Emission South China University of Technology Guangzhou 510640 China; ^5^ Shenzhen Institute of Aggregate Science and Technology School of Science and Engineering The Chinese University of Hong Kong Shenzhen, 2001 Longxiang Boulevard, Longgang District, Shenzhen City Guangdong 518172 China

**Keywords:** activated alkyne, autonomous material, damage visualization, metal‐free polymerization, microcapsule

## Abstract

The development of autonomous materials with desired performance and built‐in visualizable sensing units is of great academic and industrial significance. Although a wide range of damage indication methods have been reported, the “turn‐on” sensing mechanism by damaging events based on microcapsule systems, especially those relying on chemical reactions to elicit a chromogenic response, are still very limited. Herein, a facile and metal‐free polymerization route with an interesting reaction‐induced coloration effect is demonstrated. Under the catalysis of 1,4‐diazabicyclo[2.2.2]octane (DABCO), the polymerizations of difunctional or trifunctional activated alkynes proceed very quickly at 0 °C in air. A series of polymers composed of stereoregular enyne structure (major unit) and divinyl ether structure (minor unit) are obtained. Both the catalyst and monomers are colorless while the polymerized products are deep‐colored. This process can be applied for the damage visualization of polymers using the microencapsulation technique. Microcapsules containing the reactive alkyne monomer are prepared and mixed in a DABCO‐dispersed polymer film. Both the external and internal damage regions of this composite film can be readily visualized once the reaction is initiated from the ruptured microcapsules. Moreover, the newly formed polymer automatically seals the cracks with an additional protection function.

## Graphical abstract

This work reports an efficient metal‐free polymerization of activated alkyne as a new turn‐on indication system for the autonomous visualization of mechanical damage in polymers. By dispersing the colorless monomer‐loaded microcapsules and 1,4‐diazabicyclo[2.2.2]octane catalyst in polymer films, the damage can be readily visualized once the reaction is initiated from the ruptured microcapsules.

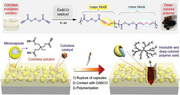

## 1 Introduction

Visualization of mechanical damage in polymers and composite materials is of great significance in both fundamental study and technological applications. Although polymers such as protective polymer coatings are designed to be as robust as possible, they are susceptible to damage in the form of small cracks that are often difficult to be detected during use. These small‐scale crack damage could grow into macro‐damage under excess mechanical load and finally compromise the mechanical integrity and materials performance of polymers.^[^ ^1^ ^]^ Therefore, it is highly desirable to develop built‐in indicators in polymeric materials that can reveal mechanical damage before catastrophic material failure or malfunction occurs. Toward this goal, a variety of damage indication systems with mechanically triggered color change in polymers have been reported. For example, by chemically incorporating mechano‐responsive small molecules or the so‐called mechanophores into polymer backbones or cross‐linker units, localized mechanochromism or mechano‐induced chemiluminescence can be achieved based on the mechanochemistry of mechanophores under large strains.^[^ ^2^ ^]^ One major limitation of this inherent type of self‐reporting materials is their complicated chemical synthesis. Physical blending of aggregachromic chromophores in polymer matrices can also achieve mechanochromic responses.^[^ ^3^ ^]^ This extrinsic approach is easy to perform but is difficult to generalize. A versatile and simple alternate strategy for damage visualization is incorporating indicator‐containing microcapsules or hollow fibers in polymer matrices.^[^ ^4^ ^]^ Such additives with fluid payload can be readily incorporated in diverse polymers of interest by simple mixing. No chemical modification is needed. The encapsulated indicators are mainly solutions of dyes or fluorescent materials that can be physically or chemically activated upon release from the microcapsules or fibers under damage. The mechanochromic effects can be realized with different activation schemes, mainly including the simple release of an encapsulated dye,^[^ ^5^ ^]^ interactions of payloads from two separate reservoirs,^[^ ^6^ ^]^ aggregation‐induced optical changes,^[^ ^7^ ^]^ and “turn‐on” mechanism based on the interactions between the released compounds and the matrix.^[^ ^8^ ^]^ Among them, the former three approaches have been widely investigated and can further achieve dual‐functions of self‐reporting and self‐repairing by the simultaneous encapsulation of indicator and healing agent into one microcapsule or by the combination of two types of single‐function microcapsules together in polymer matrix.^[^ ^9^ ^]^ By contrast, the “turn‐on” mechanism‐based encapsulated systems, especially those relying on chemical reactions to elicit a chromogenic response, are less reported.

An early and typical example of capsule‐based system showing “turn‐on” mechanism for damage visualization of polymers was reported by Moore and co‐workers.^[^ ^8c^ ^]^ The self‐reporting function was achieved by a ring‐opening metathesis polymerization (ROMP)‐based chromogenic assay. The ROMP of the light yellow 1,3,5,7‐cyclooctatetraene (COT) can efficiently form intensely colored polyacetylene in the presence of the lime green Grubbs‐Love ruthenium catalyst. The COT‐containing microcapsules and catalyst were dispersed in polymers. When the composite was scratched, an instant color change was observed in the damaged region due to the ROMP of the released COT monomer in contact with the dispersed catalyst in polymer matrix. Compared to the “turn‐on” mechanism based on the physical activation, the polymerization‐triggered color change is potential to benefit the autonomous self‐protection of polymeric materials by filling the cracks by newly formed polymers. Our research group has been working on the exploration of new alkyne‐based polymerization methods toward functional polymers for decades.^[^ ^10^ ^]^ A large variety of polymerization reactions of terminal and internal alkynes have been successfully developed from alkyne‐based organic reactions, generating diverse polymers with unique structures and advanced functionalities.^[^ ^11^ ^]^ During the investigation course of organobase‐catalyzed polymerizations of activated alkynes and diols,^[^ ^12^ ^]^ we stumbled across an unusual chromogenic phenomenon upon adding the basic catalyst into the solution of monomer mixture. The chromogenic effect was much more remarkable when the basic catalyst was added into the colorless solution of mere alkynoate monomer, producing deep‐colored insoluble solids or gels within seconds (Figure S1, Supporting Information). We speculated that the alkynoate monomer might have undergone a polymerization reaction under such conditions. If so, the interesting chromogenic effect of such polymerizations might be applied for autonomous damage visualization of polymers to enrich the “turn‐on” mechanism based on the chemical activation.

**Scheme 1:**
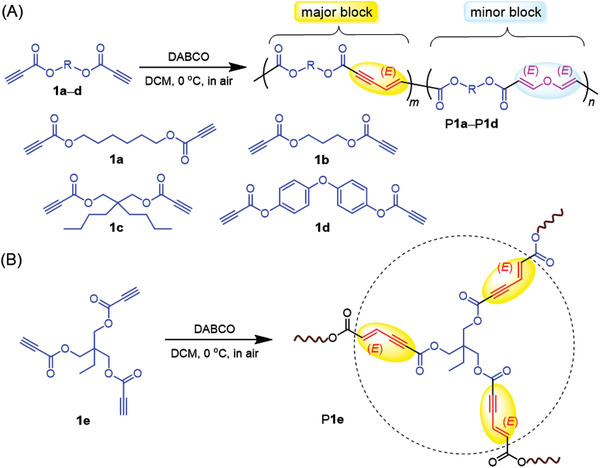
Organocatalytic polymerizations of A) dipropiolates and B) tripropiolates toward stereoregular polymers.

Although propiolates have been reported to show high reactivity in basic conditions to afford stereoregular dimerization products,^[^ ^13^ ^]^ the possibility of developing base‐catalyzed polymerizations of a single component of alkynes has rarely been explored. By investigating the effects of different reaction parameters and carefully analyzing the product structures, herein we successfully developed a metal‐free and efficient polymerization route of alkynoate monomers (**Scheme** **1**). In the presence of a commonly used organic base 1,4‐diazabicyclo[2.2.2]octane (DABCO), the polymerizations of colorless difunctional or trifunctional activated alkynes proceeded very quickly at 0 °C in air, producing colored polymers with a high molecular weight of up to 29 800 for the soluble part. The polymer structures were dominated by enyne units, whereas the divinyl ether units were also formed as the minor block due to the intervention of water in the reaction. It is worth noting that both the enyne and divinyl ether units were formed in a stereoregular way. Compared to the previously reported ROMP‐based chromogenic assay and traditional coupling polymerizations of alkynes,^[^ ^8^, ^14^ ^]^ which often require the use of transition metal catalysts, DABCO catalyst has the advantages of colorless, stable, low cost, easy accessibility, environmentally friendly, mild reaction conditions, etc. Based on the core–shell microcapsulation technique, we applied the chromogenic effect of this polymerization for the design of autonomous self‐reporting polymer coatings, and the newly formed polymer might provide additional self‐protection function to the smart coatings.

## 2 Results and Discussion

### 2.1 Organobase‐Catalyzed Polymerizations of Activated Alkynes

Monomers **1a–1e** used in this work are readily accessible and all of them are colorless and stable under ambient conditions. They can be easily synthesized through the condensation reactions of alcohols or phenols with propiolic acid.^[^ ^12^, ^15^ ^]^ The polymerizability of alkynoate monomers in basic conditions was first investigated using hexane‐1,6‐diyl dipropiolate (**1a**) as the model monomer. On the first try, the polymerization was carried out at 0 °C under a nitrogen atmosphere by dropwise adding 0.5 mL DCM solution of DABCO (10 mol%) into 0.5 mL DCM solution of **1a** (0.2 mmol). The reaction mixture immediately turned to brown upon adding the DABCO solution and the appearance color became darker and darker with the gradual addition of DABCO. After reacting for 1 h, the polymerization was stopped by adding the diluted reaction mixture into CHCl_3_/*n*‐hexane mixture (1/7, v/v) via a silica gel‐filled dropper to precipitate the polymeric product. After being filtered and dried, polymer P**1a** was obtained as brownish red solid powders in a good yield of 79% (**Table** **1**, entry 1). This polymeric product was partially soluble in common organic solvents and the *M*_n_ and *M*_w_ of the soluble part was measured to be 17 700 and 27 100, respectively.

**Table 1:** Optimization of the polymerization conditions of monomer **1a**

Entry^a)^	*T* [°C]	Concentration of **1a** [m]	DABCO [mol%]	Yield [%]	*M*_w_ ^b)^	*M*_w_/*M*_n_ ^b)^	*S* ^c)^
1^d)^	0	0.2	10	79	27 100	1.5	Δ
2	0	0.2	10	81	34 100	1.9	Δ
3	−40	0.2	10	27	28 600	1.6	Δ
4	25	0.2	10	Gelled within 0.5 h	×		
5	0	0.1	10	72	11 400	1.4	Δ
6	0	0.05	10	25	2900	1.1	Δ
7	0	0.3	10	Gelled within 10 min	×		
8	0	0.2	15	84	29 800	1.4	Δ
9	0	0.2	20	85	22 900	1.4	Δ
10	0	0.2	5	57	19 700	1.3	Δ
11	0	0.2	2.5	5	2900	1.1	Δ

^a)^ Unless otherwise noted, the polymerizations were carried out in anhydrous DCM in air for 1 h;

^b)^ Determined by GPC in THF on the basis of a linear polystyrene calibration;

^c)^ Solubility (*S*) tested in common organic solvents, such as DCM, chloroform, and THF: Δ = partially soluble, × = insoluble. The GPC results are for the soluble part;

^d)^ Polymerization results obtained under nitrogen atmosphere.

To simplify the operation procedures, we then tried to polymerize **1a** in air and the other reaction parameters were kept unchanged. As shown in entry 2 of Table 1, P**1a** was produced in a relatively higher yield of 81% in air and the molecular weight of the soluble part was also increased. The following polymerizations were thus conducted in air. As summarized in Table S1 (Supporting Information), changing the charging sequence or the states of reactants greatly affected the polymerization efficiency. When the DCM solution of **1a** (0.5 mL) was added dropwise into the DCM solution of DABCO (0.5 mL) in air, only 41% polymeric product was obtained. The reaction yield obtained by adding 1 mL of **1a** solution to DABCO powder and adding the DABCO solution to **1a** powder was 61% and 70%, respectively. Directly mixing the powder of 0.02 mmol **1a** and 10 mol% of DABCO at 0 °C gave a similar yield of 73%. When 50 mol% DABCO was used to mix with **1a** or the powder was mixed at 25 °C, a high yield of 96% could be achieved, but the obtained products were insoluble in common organic solvents. The solvent effect was then investigated at 0 °C under air by adding the DCM solution of 10 mol% DABCO to **1a** solution (Table S2, Supporting Information). Among the tested solvents, including DCM, DCM/water mixtures with different volume ratio, THF, THF/water mixture, DMF, DMSO, 1,4‐dioxane, hexyl acetate, ethyl phenylacetate (EPA), acetonitrile, and toluene, the best results were obtained from the polymerization in DCM considering both the isolated yield and the solubility of the polymeric product. Increasing the reaction temperature or monomer concentration can promote the polymerization of **1a** but led to the formation of insoluble gel, whereas lowering the temperature or concentration or shortening reaction time obviously reduced the reaction yield (Table 1, entries 2–7 and Table S3, Supporting Information). Therefore, 0 °C, 0.2 m, and 1 h were the preferred reaction parameters for producing partially soluble polymers with good efficiency.

The influence of catalyst on the polymerization was then studied. The polymerization results using different amount of DABCO suggested that the presence of 15 mol% DABCO can simultaneously improve the reaction yield and the molecular weights of the soluble polymer (Table 1, entries 8–11). When DABCO was replaced by other another commonly used organic bases, the polymerization results were significantly different. As depicted in Table S4 (Supporting Information), the addition of DCM solution of 4‐dimethylaminopyridine (DMAP) into **1a** solution also produced polymeric product as brownish red powder, but the polymer structure was undesired and unidentified. The reaction mixture turned into deep brownish red in the presence of 15 mol% trimethylamine, but no polymeric product was isolated after reacting for 1 h. When 15 mol% pyridine, 4‐picoline, or 1,8‐diazabicyclo[5.4.0]undec‐7‐ene (DBU) was used as catalyst, no remarkable color change occurred for the reaction mixture and no polymeric product was obtained within 1 h. Increasing the amount of trimethylamine, pyridine, and 4‐picoline resulted in the formation of brownish red gel, while the polymerization remained inert even in the presence of 10 equivalent of DBU. Inorganic bases such as NaOH, Na_2_CO_3_, and CH_3_COONa were also tested to initiate the polymerization. However, none of them produced polymeric products possibly due to their insolubility in DCM solvent. Therefore, 15 mol% DABCO was adopted as the optimal catalyst condition.

To investigate the monomer scope of this polymerization strategy, the polymerization activities of other activated alkynes were examined. As summarized in Table S5 (Supporting Information), the polymerization of propane‐1,3‐diyl dipropiolate (**1b**) can produce polymeric product in a yield of 49% and 84% after reacting for 1 and 2 h, respectively. The low molecular weights of the soluble part might result from the poor solubility of P**1b**. The activated dipropiolate monomer with branched alkyl groups (**1c**) merely generated a trace amount of product under the optimized conditions. By increasing the monomer concentration from 0.2 to 0.4 m, P**1c** was isolated in a yield of 9%. Compared to the aliphatic alkynes, the polymerization of aromatic activated alkyne proceeded very quickly. Insoluble gelatinous polymer was immediately formed upon adding DABCO catalyst at a monomer concentration of 0.2 or 0.1 m. Decreasing the monomer concentration to 0.05 m also led to the formation of insoluble gel within 2 min. A partially soluble polymer (P**1d**) was finally obtained by simultaneously decreasing the monomer concentration and catalyst loading. To facilitate the practical applications of this polymerization, we also explored its applicability in the preparation of hyperbranched polymer. The reaction mixture of the triyne monomer (**1e**) became gelled within seconds at a monomer concentration of 0.2 or 0.1 m. The polymerization of **1e** at a concentration of 0.05 m gave the hyperbranched polymer P**1e** as brownish red powder in a yield of 97%. These results suggested that the organocatalytic polymerizations of activated alkynes were facile and efficient at mild conditions, and showed an interesting reaction‐induced chromogenic response.

### 2.2 Structural Characterization of the Polymers

**Scheme 2:**
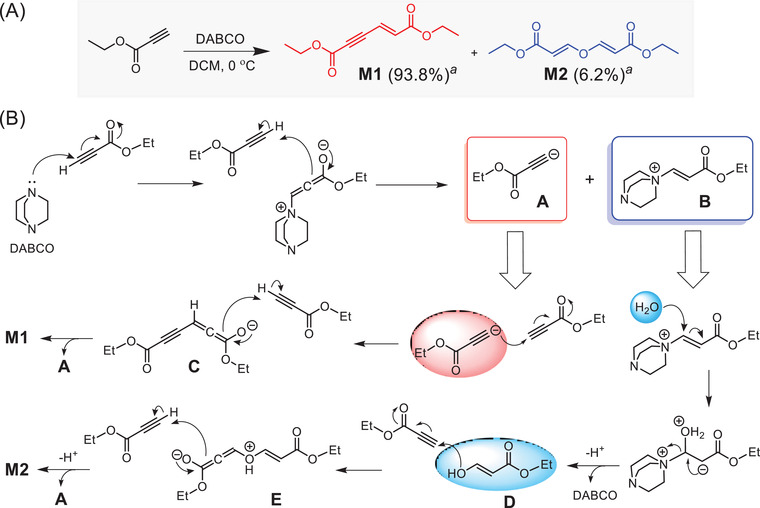
A) Synthetic route to model compounds **M1** and **M2**. B) Proposed mechanism for the formation of **M1** and **M2**. ^a^ NMR yield.

To assist the structural characterization of the obtained polymers, we conducted a model reaction of ethyl propiolate at 0 °C in air (**Scheme** **2**A).^[^ ^13a^ ^]^ After careful purification, the main product of diethyl (*E*)‐hex‐2‐en‐4‐ynedioate (**M1**) was collected as yellow oil in an NMR yield of 93.8%. The structure of **M1** was confirmed by HRMS, FTIR, and NMR analysis (see the Supporting Information for details). It is worth mentioning that a side product (**M2**) was also isolated as white solid powders in an NMR yield of 6.2%. The ^1^H NMR, ^13^C NMR, ^1^H‐^1^H COSY, and FTIR results shown in Figures S2–S4 (Supporting Information) clearly revealed that **M2** is diethyl 3,3″‐oxy(2*E*,2″*E*)‐diacrylate. The formation of this stereoregular symmetrical ether indicated the intervention of water in the reaction.^[^ ^16^ ^]^ The little amount of water in the reaction system may come from the moisture in the air or the hygroscopic DABCO. The proposed mechanism for the simultaneous formation of **M1** and **M2** was provided in Scheme 2B. First, the DABCO abstracts the acidic hydrogen of ethyl propiolate to give the anionic intermediate **A** and the cationic vinyl ester (intermediate **B**). The subsequent nucleophilic attack of A on another ethyl propiolate molecule affords the allene intermediate **C**, which then undergoes protonation to form the *E*‐enyne diester (**M1**). In the presence of water, ethyl (*E*)‐3‐hydroxyacrylate (intermediate **D**) could be generated by attacking the propiolate or intermediate B with water (hydroxide) as the nucleophile. Therefore, it is likely to afford the divinyl ether (**M2**) product through the reaction of intermediate **E** with another molecule of ethyl propiolate. The stereospecificity of the reaction could be attributed to the residue coordination between the DABCO cation and the acetylenic anion (**A**).^[^ ^13^, ^17^ ^]^

Based on the characterization results of model compounds **M1** and **M2**, we then carefully analyzed the structural composition of the obtained polymers. The FTIR and NMR spectra of polymer P**1a** are discussed here as an example. As shown in Figure S4 (Supporting Information), the stretching vibration of ≡C—H of **1a** disappeared after polymerization, whereas the C≡C and C═O stretching peak of **1a** at 2110 and 1694 cm^−1^ shifted to 2220 and 1708 cm^−1^, respectively, in the FTIR spectrum of P**1a**. Meanwhile, a new absorption peak emerged at 1618 cm^−1^, which was associated with the stretching vibration of the newly formed C═C group. These results revealed the occurrence of the polymerization. The same conclusion can also be drawn from the FTIR spectra of other polymers (Figures S5–S8, Supporting Information).

**Figure 1:**
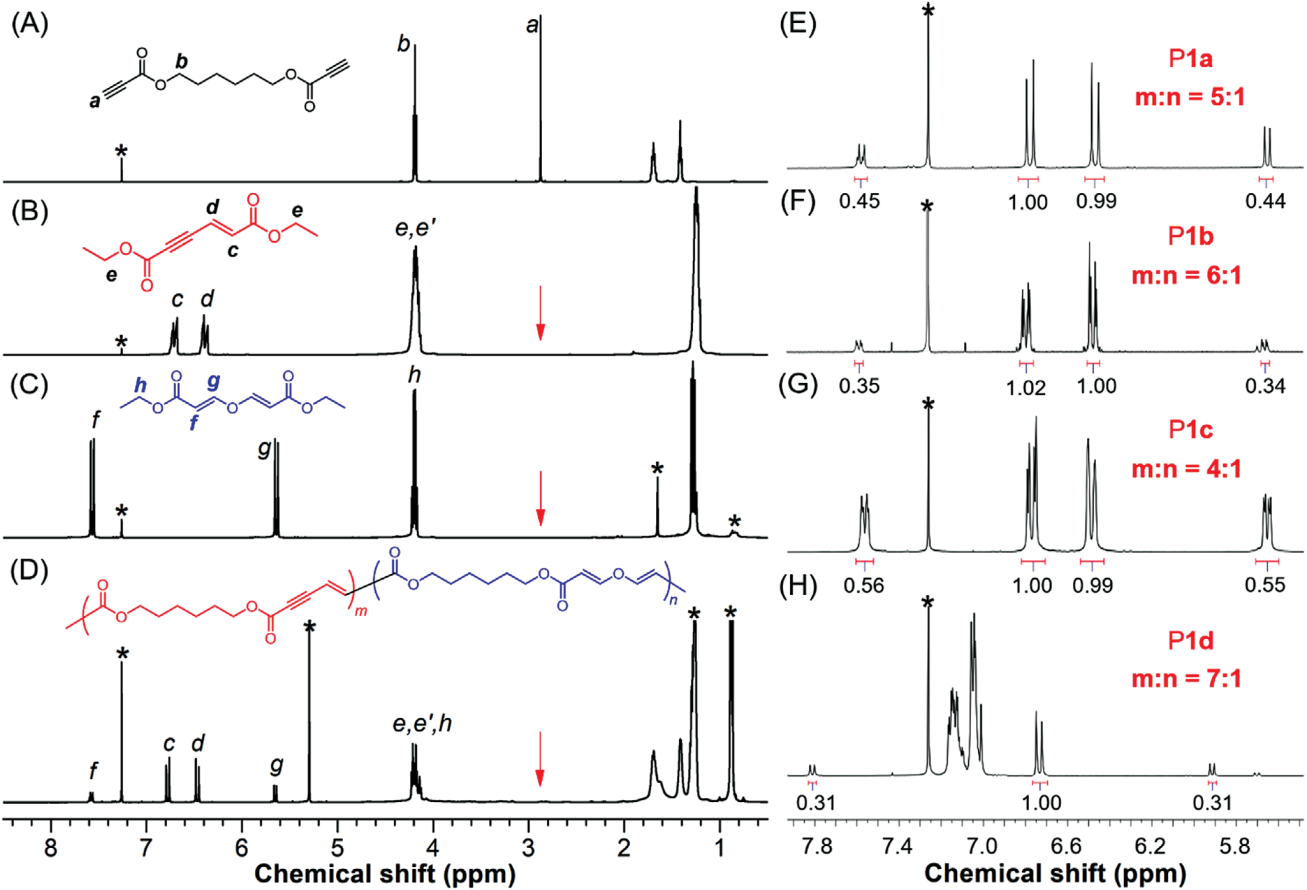
A‒D) ^1^H NMR spectra of monomer **1a**, model compound **M1**, model compound **M2**, and polymer P**1a** in chloroform‐*d*. E‒H) Enlarged ^1^H NMR spectra of P**1a**, P**1b**, P**1c**, and P**1d** in chloroform‐*d*. The solvent peaks are marked with asterisks.

More detailed information on the polymer structures was obtained from the NMR analysis. As depicted in **Figure** **1**A–D, the ^1^H NMR spectra of model compounds and P**1a** showed no resonance peak related to the ethynyl proton of **1a** at *δ* 2.88. Instead, four new peaks were observed at *δ* 7.58, 6.78, 6.48, and 5.65 in the spectrum of P**1a**. Compared with the^1^H NMR spectrum of **M1** and **M2**, the peaks at *δ* 6.78 and 6.48 can be readily assigned to the resonance signals of the vinyl protons of the *E*‐enyne diester structure in “c” and “d” position, respectively. The two small peaks at *δ* 7.58 and 5.65 were associated with the resonances of the vinyl protons in “f” and “g” positions of the divinyl ether structure. Therefore, the structure of P**1a** obtained in air was composed of both the enyne diester and the divinyl ether unit. The number ratio of enyne unit to ether unit (*m*:*n*) was estimated to be 5:1 by calculating the integration of the peak at *δ* 7.58 and 6.78 (Figure 1E). Additionally, we examined the ^1^H NMR spectrum of the polymeric product obtained under a nitrogen atmosphere (Figure S9, Supporting Information), which also showed resonance peaks related to the divinyl ether structure (*m*:*n* = 6:1). This result implied that the divinyl ether unit might be generated from the reaction of residual monomers or the end groups of polymers with water during the post‐treatment procedures. The ^1^H NMR results shown in Figure 1F–H and Figures S10–S12 (Supporting Information) revealed that P**1b**–P**1d** also consist of both the enyne diester and the divinyl ether units with enyne as the major block. P**1e** contains almost only enyne structure as indicated by the ^1^H NMR spectrum (Figure S13, Supporting Information). The addition of a large amount water into the reaction solvent greatly impeded the polymerization possibly due to the poor water solubility of monomers (Table S2, Supporting Information, entries 2, 3, and 5), and the obtained polymer structure was still dominated by the enyne unit (Figure S14, Supporting Information). Therefore, the polymerization of activated diynes with water was much less efficient than the self‐coupling polymerizations of activated diynes.

The ^13^C NMR results further verified the polymer structures. As shown in Figure S15 (Supporting Information), the peaks of the C≡C carbon atoms of **1a** completely disappeared in the ^13^C NMR spectrum of P**1a**. The carbonyl carbon (“c” position) of **1a** at *δ* 152.89 was transformed into three new peaks at *δ* 166.29, 164.89, and 153.35 after the polymerization. Based on the ^13^C NMR spectra of model compounds, the peaks at *δ* 164.89 and 153.35 were assigned to the “e” and “f” carbonyl carbons of the enyne diester unit, respectively, while the peaks *δ* 166.29 should arise from the carbonyl carbons (“l” position) of the divinyl ether unit. Meanwhile, the characteristic peaks associated with the C═C and C≡C carbon atoms in model compounds were all observed in the polymer spectrum. The ^1^H and ^13^C NMR spectra of polymers largely resemble those of **M1** and **M2**, indicating that this polymerization method is highly stereoselective. Almost no resonance peaks related to stereoisomeric structures were detected in the characterization results of polymers (Figure S16, Supporting Information). Similar to the model compounds, the C═C bonds in the enyne and vinyl ether units should also adopt an *E*‐configuration. These results clearly demonstrated that the obtained polymers have the exact structures as depicted in Scheme 1.

On the basis of the mechanism of the model reaction (Scheme 2B), we proposed a plausible reaction mechanism for this polymerization. As shown in Scheme S1 (Supporting Information), DABCO first reacts with activated diyne to produce the dianionic intermediate (I) and the dicationic vinyl ester intermediate (II). Then the dianionic intermediate further reacts with other alkyne monomers to initiate the chain propagation process and finally form the major block of poly(1,3‐enyne). The minor block of poly(vinyl ether) may be generated by the nucleophilic reaction of water with the intermediate II (Path A, Scheme S2, Supporting Information) or by the direct attack of water to the diyne monomer (Path B, Scheme S2, Supporting Information). As the nucleophilic ability of water is obviously weaker than the dianionic intermediate I, it is reasonable that the copolymer structures are dominated by the 1,3‐enyne unit.

### 2.3 Thermal Stability and Color Property of Polymers

The obtained polymers possessed good thermal and morphological stability. As shown in Figure S17 (Supporting Information), the decomposition temperature (*T*_d_) of the polymers at 5% weight loss ranged from 290 to 318 °C. DSC results suggested that P**1b** and P**1e** possessed a glass transition temperature (*T*_g_) of 120 and 122 °C, respectively (Figure S18, Supporting Information), while the *T*_g_ values of P**1a** and P**1d** were somehow difficult to be detected by DSC.

**Figure 2:**
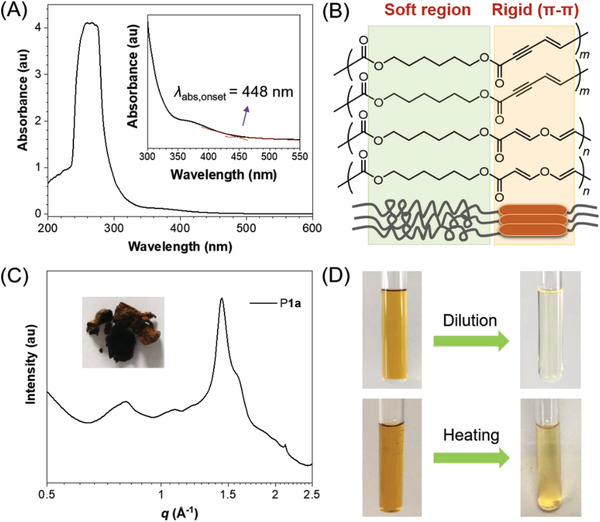
A) UV–vis absorption spectra of P**1a** in CDCl_3_ solutions and its amplified part. B) Schematic description of the possible interactions between P**1a** chains. C) Wide angle X‐ray scattering profile of P**1a**. Inset: the picture of P**1a** solid. D) Pictures showing the color change of the THF solution of P**1a** before and after heating at 68 °C for 1 h or after dilution.

To investigate the reaction‐induced chromogenic response of this polymerization, the color properties of these polymers were then investigated. The dilute solution of P**1a** exhibited a maximum and onset absorption (*λ*_abs,onset_) at 266 and 448 nm, respectively, showing little absorption in the visible region (**Figure** **2**A). This is understandable as the structure of P**1a** possesses weak electronic conjugation. However, the solid powder of P**1a** showed an unexpected brownish red color (Table S6, Supporting Information, entry 1). Similar brownish red appearance was also observed in P**1e** powders (Table S6, Supporting Information, entry 5). By contrast, polymers P**1b** with shorter alkoxyl spacer, P**1c** with bulky side groups, and P**1d** with phenyl rings showed yellow appearance in solid states (Table S6, Supporting Information, entries 2–4). These results suggested that the color properties were influenced by polymer structures. It is worth noting that an immediate color change from colorless to brown red was observed whether the DABCO was added into the DCM solution of ethyl propiolate (monofunctional alkyne) or monomer **1a**‒**1e** (di‐/trifunctional alkyne). After the reaction mixture was purified by silica gel or was treated by dilute hydrochloric acid, the color of the mixture solution and the obtained products became obviously lighter. This phenomenon indicated that the presence of DABCO catalyst greatly contributed to this chromogenic effect. The coloration effect of different catalysts shown in Table S4 (Supporting Information) also supported this conclusion. The interaction between the activated alkynes and DABCO catalyst might lead to the formation of some certain colored substances or complexes during the reaction process (Scheme 2 and Schemes S1 and S2, Supporting Information), and most of the colored intermediates or complexes could be absorbed by silica gel in the post‐treatment procedures. Moreover, there might exist strong inter‐/intra‐chain interactions in P**1a** and P**1e** in solid states, which could trap some of the possible colored intermediates and meanwhile generate through‐space conjugation.^[^ ^18^ ^]^ Taking P**1a** as an example, the co‐existence of flexible hexyloxyl segments and relatively rigid enyne and vinyl ether units might facilitate the formation of strong *π*–*π* interactions between the C═C and C≡C units in solid states (Figure 2B). The wide angle X‐ray scattering pattern measured by a synchrotron suggested that the deep‐colored solid powder of P**1a** is mainly in amorphous state, but there is a certain degree of crystallization due to the oriented stacking in some directions (Figure 2C). Besides, the appearance color of P**1a** solution became shallow after being diluted or heated (Figure 2D), which also supported our abovementioned speculation.

### 2.4 Application of the Polymerization in Self‐Reporting Coatings

**Figure 3:**
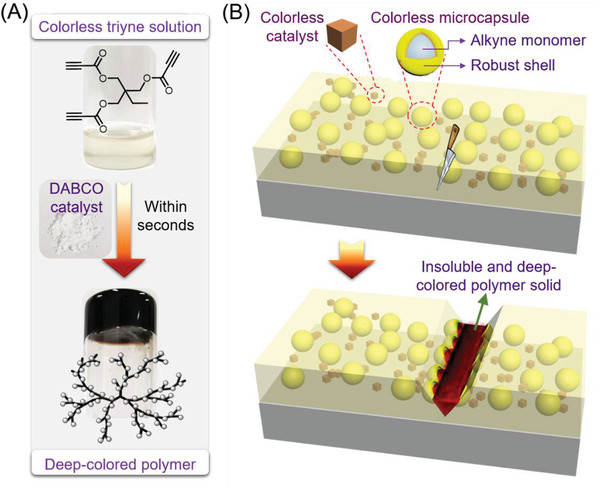
A) Pictures showing the instantaneous color change from the colorless ethyl phenylacetate solution of triyne monomer to the deep‐colored hyperbranched polymer after adding the colorless DABCO catalyst. B) Schematic description of autonomous self‐reporting coatings using core–shell microcapsules based on the color change of the organocatalytic polymerizations of activated alkynes.

Monomers **1a**–**1e** used in the polymerization are colorless liquid or white powder and the DABCO crystals are also colorless. However, the untreated polymeric products are deep‐colored. Attracted by such a remarkable color change before and after polymerization, we then tried to apply this facile and efficient polymerization for the autonomous visualization of mechanical damage using core–shell microcapsules. EPA is a commonly used solvent for the fabrication of microcapsules. As depicted in **Figure** **3**A, the colorless EPA solution of the triyne monomer (**1e**) was immediately transformed into intensely colored polymer gel upon adding the colorless DABCO catalyst. By dispersing the colorless DABCO catalyst and microcapsules containing the EPA solution of activated alkyne monomer in a polymer matrix, smart coatings with autonomous self‐reporting function could be produced. As illustrated in Figure 3B, when cracks form in the matrix wherever mechanical damage occurs, the microcapsules will be ruptured to release the alkyne monomer into the crack region through capillary action. Polymerization of the activated alkyne monomer is then triggered on contact with the embedded DABCO catalyst in the matrix, generating insoluble and intensely colored polymeric products. In this way, the mechanical damage can be readily detected based on the color contrast. Besides, the crack region might be filled and protected by the resulting polymer.

**Figure 4:**
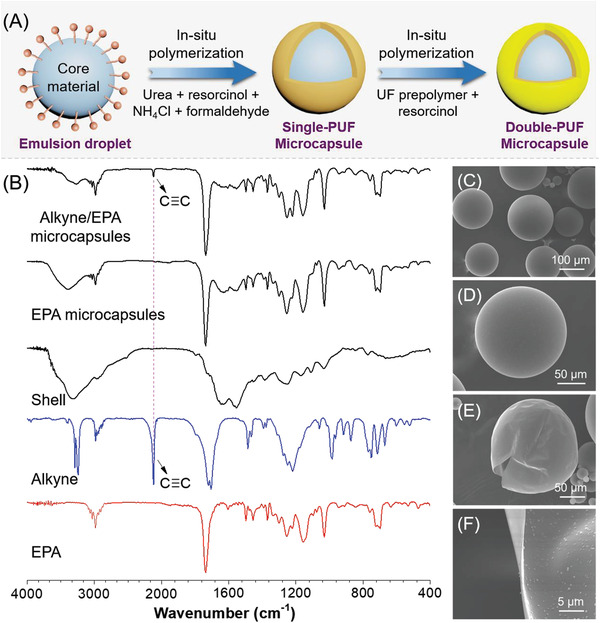
A) Schematic description of the fabrication procedure of microcapsules. B) IR spectra of alkyne/EPA microcapsules, EPA microcapsules, shell, alkyne monomer, and pure EPA. SEM images showing C,D) the intact morphology, E) ruptured morphology, and F) shell wall profile of alkyne/EPA microcapsules. Alkyne content = 1.0 wt%.

To demonstrate our design concept, stable double‐layered microcapsules containing the EPA solution of **1e** (referred to as alkyne/EPA microcapsules) were first prepared using a two‐step process based on the urea‐formaldehyde in situ polymerization.^[^ ^8^, ^19^ ^]^ As schematically illustrated in **Figure** **4**A, in the first step, the inner poly(urea‐formaldehyde) (PUF) shell of the microcapsule was formed via an in situ interfacial polymerization in an oil‐in‐water emulsion. Into the aqueous solution (0.5 wt%) of poly(ethylene‐alt‐maleic anhydride) emulsifier were sequentially added urea, resorcinol, and NH_4_Cl. Then the alkyne/EPA solution was added dropwise into this aqueous solution at a pH of 3.5 under agitation for 15 min to form the emulsion microdroplets. Subsequently, formaldehyde was added into the mixture and the temperature was raised to 55 °C to initiate the interfacial polymerization. Single‐layered PUF microcapsules were produced after reacting for 4 h. In the second step, the outer PUF shell was synthesized by mixing the mixture from the first step with the presynthesized UF prepolymer and resorcinol. The pH was adjusted to 3 and the mixture was allowed to react at 55 °C for another 3 h. Finally, double‐layered microcapsules containing the EPA solution of alkyne were obtained after being rinsed with deionized water several times and dried in air overnight. Following the aforementioned procedures, a series of alkyne/EPA microcapsules were prepared from alkyne/EPA solutions with different alkyne contents including 0.5, 1.0, and 5.0 wt%. Besides, microcapsules containing pure EPA solvent as the core material (referred to as EPA microcapsules) were also prepared for control experiments.

The composition and morphologies of the obtained microcapsules were analyzed using FTIR and SEM, respectively. As summarized in Figure 4B, the characteristic peak associated with the stretching vibration of C≡C of **1e** was observed at 2120 cm^−1^ in the IR spectrum of alkyne/EPA microcapsules. Meanwhile, the absorption peaks related to the aromatic and aliphatic C—H stretching vibrations of EPA and **1e** at 3090–2850 cm^−1^ as well as the broad absorption band arising from the PUF shells were all detected in the spectra of alkyne/EPA and EPA microcapsules. These results indicated that the core materials of alkyne/EPA or EPA were successfully encapsulated into the microcapsules. The SEM images of intact microcapsules shown in Figure 4C,D and Figures S19 and S20 (Supporting Information) demonstrated that the obtained alkyne/EPA microcapsules and EPA microcapsules are spherical in shape with smooth outer surfaces and good morphology uniformity. The mean diameter of the microcapsules was measured to be 106 µm (sample size = 200) and the corresponding size distribution was shown in Figure S21 (Supporting Information). The core–shell structures of these microcapsules are confirmed by the morphologies of ruptured microcapsules (Figure 4E and Figure S19C,F,J, Supporting Information), and the polymer shell wall thickness was around 260 nm (Figure 4F). The thermogravimetric analysis results suggested that the encapsulated EPA and alkyne possessed higher thermal degradation temperatures than those of the naked ones due to the presence of double‐layer PUF/PUF shells (Figure S22, Supporting Information). It is worth noting that the SEM results shown in Figure S20 (Supporting Information) were recorded on microcapsules that had been placed for over one year, which is indicative of the excellent morphological stability of the microcapsules at ambient conditions.

**Figure 5:**
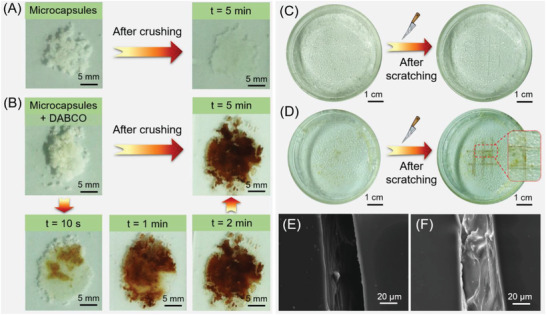
A) Photographs of alkyne/EPA microcapsules without catalyst before crushing and after crushing between glass slides for 5 min. B) Photographs of alkyne/EPA microcapsules mixed with 10 wt% DABCO catalyst before crushing and after crushing between glass slides for different time. Photographs of PVA films containing C) only 15 wt% alkyne/EPA microcapsules and D) 15 wt% alkyne/EPA microcapsules and 5 wt% DABCO catalyst before and after being scratched with a utility knife for 10 min. SEM images of the scratched region of E) the control PVA film containing 5 wt% DABCO catalyst and F) the PVA film containing 15 wt% alkyne/EPA microcapsules and 5 wt% DABCO catalyst after being scratched for 12 h. Alkyne/EPA microcapsules were prepared using alkyne/EPA solution containing 5 wt% alkyne. The amount of catalyst was relative to the dosage of the microcapsules.

We next investigated the potential application of the alkyne/EPA microcapsules for mechanical damage visualization via color change. When the alkyne/EPA microcapsules were crushed in the absence of DABCO catalyst by compression between two glass slides, colorless core materials were efficiently released but no color change was observed (**Figure** **5**A). The contact between intact alkyne/EPA microcapsules and DABCO catalyst also led to no color change (Figure 5B, Figures S23 and S24, Supporting Information). However, an evident release of core materials accompanied with an immediate color change occurred upon crushing the alkyne/EPA microcapsules (alkyne content = 5 wt%) in the presence of 10 wt% DABCO catalyst (Figure 5B). The color became darker and darker and meanwhile the released liquid core materials were gradually solidified as time went on. After placing for 5 min, the mixture became dark red solids. This color change is consistent with the phenomenon observed during the polymerization. The results shown in Figure S23 (Supporting Information) suggested that the microcapsules prepared from alkyne/EPA solution with an alkyne content of 5.0 wt% showed much better damage indication capability than microcapsules with 0.5 and 1.0 wt% alkyne. Obvious red color can still be observed after reducing the dosage of catalyst from 50 to 10 wt% (Figure S24, Supporting Information). To evaluate the sensitivity of this mechanochromic sensing system, quantitative crushing experiments were then conducted. Figure S25 (Supporting Information) shows the mechanchromic response of the mixture of alkyne/EPA microcapsules (alkyne content = 5 wt%) and 10 wt% DABCO catalyst under different pressure. The mixture shown in Figure S25A–C (Supporting Information) was placed between two glass slides and crushed under the compressive force of about 2, 5, and 10 N, respectively. An instantaneous color change was observed under a small force of 2 N, indicating the good mechanochromic sensitivity of the microcapsules. The higher compressive force was applied on the sample, the more evident and faster was the chromogenic response. Furthermore, UV–vis spectroscopy was used to spectroscopically characterize the color change of microcapsules. As shown in Figure S26 (Supporting Information), in the absence of the DABCO catalyst, the ruptured alkyne/EPA microcapsules absorb little light in the visible region as the onset absorption locates at about 400 nm. By contrast, a remarkable redshift in the absorption was observed after mixing the microcapsules with the catalyst, and the *λ*_abs,onset_ located at 740 nm for the first scan. The absorption spectra in the second and third scan gradually redshifted to 770 nm accompanied with stronger absorbances (each scan takes about 1 min). These spectroscopic results are consistent with the appearance color and the time‐dependent color change shown in Figure 5A,B.

Lastly, we tested the autonomous self‐reporting capabilities of the DABCO‐triggered alkyne/EPA microcapsule system in polymer coating. To homogeneously dissolve the DABCO catalyst and meanwhile minimize the leaching of hydrophobic alkyne core into the polymer matrix during coating preparation, we selected the aqueous solution of poly(vinyl alcohol) (PVA) for processing this proof‐of‐concept study. PVA films containing different amount of DABCO catalyst and alkyne/EPA microcapsules (alkyne content = 5 wt%) were prepared by casting the aqueous solution of PVA into glass petri dishes followed by sequentially adding the catalyst and microcapsules. After drying the solution in an air oven for 7 h at 60 °C, transparent PVA composite films containing microcapsules and DABCO catalyst were obtained. Control films were also prepared following similar procedures, where 1) only alkyne/EPA microcapsules were embedded, 2) only DABCO catalyst were incorporated, and 3) neither microcapsules nor catalyst were included. Details of the film preparation can be found in the Experimental Section. The obtained PVA (composite) films were manually scratched with a utility knife. As depicted in Figure 5C and Figure S27 (Supporting Information), no color change was observed in all of the control samples after being damaged over time. In contrast, the PVA film containing 15 wt% alkyne/EPA microcapsules and 10 wt% DABCO catalyst displayed obvious dark red color in the ruptured and scratched regions (Figure S28A, Supporting Information). However, at this condition, some of the microcapsules were damaged during coating preparation. To alleviate this issue, we tried to decrease the dosage of microcapsules and/or catalyst in PVA films. As depicted in Figure 5D and Figure S28B (Supporting Information), after decreasing the amount of catalyst to 5 wt%, the damage can also be clearly indicated by the color contrast in the scratched region while most of the microcapsules can remain intact before scratching. The color of the damaged areas in PVA coating continued to darken over time, and obvious solidification of the released core materials was observed within the cuts after 10 min. The damage indication effect was weakened when 10 wt% microcapsules were embedded in PVA films together with 10 or 5 wt% catalyst (Figure S28C,D, Supporting Information). Therefore, the use of 15 wt% alkyne/EPA microcapsules and 5 wt% DABCO catalyst was optimal among the tested conditions for the fabrication of autonomous self‐reporting coatings. The SEM images revealed that the crack in the PVA film where alkyne/EPA microcapsules and catalyst coexist was filled by the newly formed materials while the crack in control PVA film was empty (Figure 5E,F). This result demonstrated that the aforementioned PVA composite films possess potential self‐protection function in addition to the damage‐reporting property. Besides, the dark red color in the damaged areas was irreversible and can remain stable after placing for over one year, suggesting that this self‐reporting system can record the experienced damage of the polymer composite in the long term. Therefore, our design concept shown in Figure 3B is feasible. This reaction‐induced color change is applicable for the design of autonomous self‐reporting polymer coatings and the newly formed polymer might provide self‐protection function.

In addition to the above discussed scratch experiments, the mechanochromic response of the composite coatings under other damage modes such as impact and compressive forces was also investigated. As shown in Figure S29A (Supporting Information), the blending of 15 wt% intact alkyne/EPA microcapsules and 5 wt% DABCO catalyst in the polymer matrix led to no color change. When the polymer composite was subjected to impact force by an iron bar, the damaged areas showed immediate color change from colorless to red (Figure S29B, Supporting Information). After more impact and compressive forces were applied on the composite film by the iron bar without any external scratch, the internally damaged polymer structure can be clearly visualized by the deep red color within 1 min, while the intact areas kept colorless (Figure S29C, Supporting Information). Similar to the results of the scratch experiments, the color of the “subcutaneous” damage in the PVA coating became more evident over time (Figure S29C–G, Supporting Information). These results further demonstrated the versatility of this built‐in damage indicating system.

## 3 Conclusion

In summary, we developed a facile and efficient metal‐free polymerization method for the autonomous visualization of mechanical damage in polymeric materials. The DABCO‐catalyzed polymerizations of colorless activated alkynes proceeded quickly at 0 °C or at room temperature in air, producing a series of colored polymers with *E‐*enyne diester as the dominated structure. The divinyl ether moiety with an *E*‐configuration was also detected in polymer structures as the minor block possibly due to the intervention of water in the reaction. The obtained polymers possessed good thermal and morphological stability, and polymer P**1a** and P**1e** showed deep‐colored appearance. This reaction‐induced chromogenic effect might result from the interaction between the DABCO catalyst and the activated alkynes, and the presence of strong intra‐/inter‐chain interactions in solids of P**1a** and P**1e** could also contribute to this chromogenic response. Based on the microcapsulation technique, this polymerization system was successfully applied for the autonomous damage visualization of polymers. Stable double‐layered microcapsules containing the EPA solution of reactive triyne monomer were prepared. These colorless microcapsules showed immediate color change upon rupture in the presence of DABCO catalyst, and the color continued to darken over time. By dispersing the alkyne‐containing microcapsules and DABCO catalyst in PVA films, the external scratch and internal damage regions of the composite films can be readily visualized once the reaction is initiated from the ruptured microcapsules. Meanwhile, the cracks in the scratched PVA films were filled by the newly formed polymeric materials due to the damage‐triggered polymerization. Therefore, this work provides a promising built‐in indicating system for the design of autonomous self‐reporting polymer coatings with potential self‐protection function. With further efforts in optimizing the structures and mechanical properties of the polymeric products, this DABCO‐catalyzed polymerization might be useful in the preparation of smart coatings with dual functions of self‐reporting and self‐healing. Moreover, we envisioned that this polymerization system may be possible to be used for the generation of mechanopatterns if a large solubility difference can be achieved between the polymer matrix and the newly formed polymer in cracks. The exploration of these further applications is currently undergoing in our lab.

## 4 Experimental Section

### Polymer Synthesis

Unless otherwise noted, all the polymerization reactions were conducted under air. A typical procedure for the polymerization of hexane‐1,6‐diyl dipropiolate (**1a**) is given below as an example. Into a 10 mL reaction tube equipped with a stir bar was added **1a** (44.5 mg, 0.20 mmol). Then 0.50 mL of anhydrous DCM was injected to dissolve the monomer. Subsequently, a solution of DABCO (3.4 mg, 0.03 mmol) in 0.50 mL DCM was added dropwise to the solution of **1a**. The reaction mixture immediately turned to brown upon adding the DABCO solution and the appearance color became darker and darker with the gradual addition of DABCO. After stirring for 1 h at 0 °C in an ice‐water bath, the polymerization was stopped. The resulting solution was diluted with 2 mL of DCM and then added dropwise into 80 mL of CHCl_3_/*n*‐hexane mixture (1/7, v/v) via a dropper filled with silica gel to remove the DABCO catalyst residue. The precipitate was finally collected after filtration, and then dried under vacuum at room temperature to a constant weight. The structural characterization results are summarized as follows.

### Preparation of Microcapsules

Double‐layered microcapsules containing EPA solvent or containing the EPA solution of alkyne (**1e**) were prepared by a two‐step process based on the in situ polymerization in an oil‐in‐water emulsion.^[^ ^8^, ^19^ ^]^ Typically, 30 mL aqueous solution (0.5 wt%) of poly(ethylene‐*alt*‐maleic anhydride) (EMA, *M*_w_ = 100,000‒500,000) was prepared in the first step, into which 0.4 g urea, 0.04 g resorcinol, and 0.04 g NH_4_Cl were then added. After adjusting the pH to 3.5, 4 g EPA solution of alkyne was dispersed in the aqueous solution under an agitation rate of 500 rpm for 15 min. 1 g formaldehyde was then added into the mixture and the temperature was raised and held at 55 °C for 4 h. In the second step, urea‐formaldehyde (UF) prepolymer was first prepared by the reaction of 2.5 g formaldehyde solution, 1 g urea, and 5 g water under a pH of 8 at 70 °C for 1 h. Then the resulting mixture from the first step was mixed with the UF prepolymer and 0.6 g resorcinol. After adjusting the pH to 3, the system was left to react at 55 °C for another 3 h. The final microcapsules containing EPA solution of alkyne were rinsed with deionized water several times and dried in air overnight.

### Preparation of Polymer Films

PVA films were prepared using a casting technique. The PVA solution was prepared by dissolving PVA (20 g) in distilled water (200 mL). First, into a thermostat water bath was placed with a 500 mL three‐necked, round‐bottomed flask containing 200 mL water. Subsequently, the PVA powder was slowly added into the flask and the mixture was stirred with a digital mechanical agitator driving a two‐bladed propeller at 30 °C for about 1 h to swell the polymer. Then the temperature was gradually increased to 80 °C under stirring and this heating process took about 30 min. The mixture was then kept stirring at 80 °C for 3 h until the polymer became completely soluble. After cooling to room temperature, 10 mL of the obtained PVA solution was transferred into a glass petri dish with a diameter of 6 cm. A homogeneous PVA film was obtained after drying the glass petri dish in an air oven for 7 h at 60 °C.

The PVA films containing DABCO catalyst and microcapsules were prepared according to the following typical procedures. Into a glass petri dish containing PVA aqueous solution (10 wt%, 10 mL) was added 0.05 g DABCO catalyst. After the DABCO catalyst was completely dissolved in water, 0.15 g alkyne/EPA microcapsules with an alkyne content of 5.0 wt% was then dispersed in the aqueous solution. By drying the glass petri dish in an air oven for 7 h at 60 °C, a PVA film containing 15 wt% alkyne/EPA microcapsules and 5 wt% DABCO catalyst was obtained. For the PVA composite film used for the impact experiments, a round plastic dish with a diameter of 10 cm was used, into which 20 mL of PVA aqueous solution (10 wt%), 0.1 g DABCO catalyst, and 0.3 g alkyne/EPA microcapsules with an alkyne content of 5.0 wt% was sequentially added.

### Statistical Analysis

Quantitative data for the size distribution of microcapsules were shown as mean ± standard deviation (SD). Sample size (*n*) for each statistical analysis was described. Image J and Origin 9.0 software were used for statistical analysis.

## Conflict of Interest

The authors declare no conflict of interest.

## Supporting information

Supporting Information

## Data Availability

The data that support the findings of this study are available from the corresponding author upon reasonable request.

## References

[1] J. F. Patrick , M. J. Robb , N. R. Sottos , J. S. Moore , S. R. White , Nature 2016, 540, 363. 27974778 10.1038/nature21002

[2] a) M. Li , Q. Zhang , Y. N. Zhou , S. P. Zhu , Prog. Polym. Sci. 2018, 79, 26;

[3] a) Y. Sagara , S. Yamane , M. Mitani , C. Weder , T. Kato , Adv. Mater. 2016, 28, 1073; 26461848 10.1002/adma.201502589

[4] a) C. Calvino , C. Weder , Small 2018, 14, 1802489; 10.1002/smll.201802489 30265445

[5] a) B. Di Credico , G. Griffini , M. Levi , S. Turni , ACS Appl. Mater. Interfaces 2013, 5, 6628; 23841485 10.1021/am401328f

[6] a) D. Dohler , S. Rana , H. Rupp , H. Bergmann , S. Behzadi , D. Crespy , W. H. Binder , Chem. Commun. 2016, 52, 11076; 10.1039/c6cc05390d 27538696

[7] a) C. Calvino , A. Guha , C. Weder , S. Schrettl , Adv. Mater. 2018, 30, 1704603; 10.1002/adma.201704603 29345378

[8] a) H. Zhang , X. Zhang , C. L. Bao , X. Li , F. Duan , K. Friedrich , J. L. Yang , Chem. Mater. 2019, 31, 2611;

[9] a) Y. K. Song , T. H. Lee , K. C. Lee , M. H. Choi , J. C. Kim , S. H. Lee , S. M. Noh , Y. I. Park , Appl. Surf. Sci. 2020, 511, 145556;

[10] a) J. Z. Liu , J. W. Y. Lam , B. Z. Tang , Chem. Rev. 2009, 109, 5799; 19678641 10.1021/cr900149d

[11] a) Y. Liu , A. J. Qin , B. Z. Tang , Prog. Polym. Sci. 2018, 78, 92;

[12] a) Y. Shi , T. W. Bai , W. Bai , Z. Wang , M. Chen , B. C. Yao , J. Z. Sun , A. J. Qin , J. Ling , B. Z. Tang , Chem.‐Eur. J. 2017, 23, 10725; 28670822 10.1002/chem.201702966

[13] a) P. V. Ramachandran , M. T. Rudd , M. V. R. Reddy , Tetrahedron Lett. 2005, 46, 2547;

[14] a) U. H. F. Bunz , Chem. Rev. 2000, 100, 1605; 11749277 10.1021/cr990257j

[15] a) H. K. Li , J. Wang , J. Z. Sun , R. R. Hu , A. J. Qin , B. Z. Tang , Polym. Chem. 2012, 3, 1075;

[16] a) J. Tae , K. O. Kim , Tetrahedron Lett. 2003, 44, 2125;

[17] A. W. Mcculloch , A. G. Mcinnes , Can. J. Chem. 1974, 52, 3569.

[18] a) D. J. Ahn , J. M. Kim , Acc. Chem. Res. 2008, 41, 805; 18348539 10.1021/ar7002489

[19] H. Zhang , X. Zhang , C. L. Bao , X. Li , D. W. Sun , F. Duan , K. Friedrich , J. L. Yang , J. Mater. Chem. A 2018, 6, 24092.

