# Supplementary material for: Autonomous Visualization of Damage in Polymers by Metal‐Free Polymerizations of Microencapsulated Activated Alkynes
Source: Adv Sci (Weinh). 2022 Jan 23;9(8):2105395. doi: 10.1002/advs.202105395 (PMC8922127; doi:10.1002/advs.202105395)
Supplement: Supplementary file 1 — Supporting Information [file ADVS-9-2105395-s001.pdf]

ADVANCED SCIENCE
Open Access

Supporting Information

for *Adv. Sci.*, DOI 10.1002/advs.202105395

Autonomous Visualization of Damage in Polymers by Metal-Free Polymerizations of Microencapsulated Activated Alkynes

Ting Han, Shusheng Chen, Xinnan Wang, Xinyao Fu, Haifei Wen, Zaiyu Wang, Dong Wang, Anjun Qin*, Jinglei Yang* and Ben Zhong Tang**

ADVANCED SCIENCE
Open Access

Supporting Information

for *Adv. Sci.*, DOI: 10.1002/advs.202105395

Autonomous Visualization of Damage in Polymers by Metal-Free Polymerizations of Microencapsulated Activated Alkynes

Ting Han, Shusheng Chen, Xinnan Wang, Xinyao Fu, Haifei Wen, Zaiyu Wang, Dong Wang, Anjun Qin,* Jinglei Yang,* and Ben Zhong Tang**

Supporting Information

Autonomous Visualization of Damage in Polymers by Metal-Free Polymerizations of Microencapsulated Activated Alkynes

Ting Han, Shusheng Chen, Xinnan Wang, Xinyao Fu, Haifei Wen, Zaiyu Wang, Dong Wang, Anjun Qin,* Jinglei Yang,* and Ben Zhong Tang**

T. Han, H. Wen, D. Wang
Center for AIE Research, Shenzhen Key Laboratory of Polymer Science and Technology, Guangdong Research Center for Interfacial Engineering of Functional Materials, College of Materials Science and Engineering, Shenzhen University, Shenzhen 518060, China
E-mail: hanting@szu.edu.cn

S. Chen, J. Yang
Department of Mechanical and Aerospace Engineering, The Hong Kong University of Science and Technology, Clear Water Bay, Kowloon, Hong Kong, China
E-mail: maeyang@ust.hk

X Wang, Z. Wang, B. Z. Tang
Department of Chemistry, Hong Kong Branch of Chinese National Engineering Research Center for Tissue Restoration and Reconstruction, The Hong Kong University of Science and Technology, Clear Water Bay, Kowloon, Hong Kong, China
E-mail: tangbenz@cuhk.edu.cn

X. Fu, A. Qin, B. Z. Tang
State Key Laboratory of Luminescent Materials and Devices, Guangdong Provincial Key Laboratory of Luminescence from Molecular Aggregates, SCUT-HKUST Joint Research Institute, AIE Institute, Center for Aggregation-Induced Emission, South China University of Technology, Guangzhou 510640, China
E-mail: msqinaj@scut.edu.cn

B. Z. Tang
Shenzhen Institute of Aggregate Science and Technology, School of Science and Engineering, The Chinese University of Hong Kong, Shenzhen, 2001 Longxiang Boulevard, Longgang District, Shenzhen City, Guangdong 518172, China

Table of Contents

Experimental Section

Figure S1. Photographs showing the chromogenic phenomenon upon adding DABCO crystals into the dichloromethane solution of activated triyne.

Table S1. Preliminary Trials on the Polymerization of **1a**.

Table S2. Effect of Solvent on the Polymerization of **1a**.

Table S3. Time Course of the Polymerization of **1a**.

Table S4. Effect of Catalyst Type on the Polymerization of **1a**.

Table S5. Polymerization results of monomer **1a**–**1e**.

Figure S2. (A) ^{1}H NMR spectrum and (B) ^{13}C NMR spectrum of model compound **M2** in chloroform-*d*.

Figure S3. ^{1}H-^{1}H COSY spectrum of model compound **M2** in chloroform-*d*.

Figure S4. FT-IR spectra of (A) monomer **1a**, (B) model compound **M1**, (C) model compound **M2**, and (D) polymer P**1a**.

Figure S5. FT-IR spectra of (A) monomer **1b**, (B) model compound **M1**, (C) model compound **M2**, and (D) polymer P**1b**.

Figure S6. FT-IR spectra of (A) monomer **1c**, (B) model compound **M1**, (C) model compound **M2**, and (D) polymer P**1c**.

Figure S7. FT-IR spectra of (A) monomer **1d**, (B) model compound **M1**, (C) model compound **M2**, and (D) polymer P**1d**.

Figure S8. FT-IR spectra of (A) monomer **1e**, (B) model compound **M1**, (C) model compound **M2**, and (D) polymer P**1e**.

Figure S9. (A) General structure of the copolymers. (B-E) ^{1}H NMR spectra of (B) polymer P**1a**, (C) polymer P**1b**, (D) polymer P**1c**, and (E) polymer P**1d** in chloroform-*d*.

Figure S10. ^{1}H NMR spectra of (A) monomer **1b**, (B) model compound **M1**, (C) model compound **M2**, and (D) polymer P**1b** in chloroform-*d*.

Figure S11. ^{1}H NMR spectra of (A) monomer **1c**, (B) model compound **M1**, (C) model compound **M2**, and (D) polymer P**1c** in chloroform-*d*.

Figure S12. ^{1}H NMR spectra of (A) monomer **1d**, (B) model compound **M1**, (C) model compound **M2**, and (D) polymer P**1d** in chloroform-*d*.

Figure S13. ^{1}H NMR spectra of (A) monomer **1e**, (B) model compound **M1**, (C) model compound **M2**, and (D) polymer P**1e** in chloroform-*d*.

Figure S14. ^{1}H NMR spectrum of polymer P**1a** in chloroform-*d*. The sample was obtained from the polymerization in THF/water mixture (v/v = 1:1).

Figure S15. ^{13}C NMR spectra of (A) monomer **1a**, (B) model compound **M1**, (C) model compound **M2**, and (D) polymer P**1a** in chloroform-*d*.

Figure S16. ^{13}C NMR spectra of (A) monomer **1b**, (B) model compound **M1**, (C) model compound **M2**, and (D) polymer P**1b** in chloroform-*d*.

Scheme S1. Proposed mechanism for the formation of poly(1,3-enyne)s.

Scheme S2. Proposed mechanism for the formation of copolymers.

Figure S17. TGA thermograms of polymers recorded under nitrogen at a heating rate of 10 ^{o}C/min.

Figure S18. (A) DSC thermograms of polymers and the amplified DSC curve of (B) P**1b** and (C) P**1e** recorded under nitrogen during the second heating cycle at a heating rate of 10 ^{o}C/min.

Table S6. Appearance of Polymers P**1a**–P**1e**.

Figure S19. SEM images of intact and ruptured microcapsules containing (A-C) pure EPA solvent, (D-F) alkyne/EPA solution (0.5 wt%), and (H-J) alkyne/EPA solution (5.0 wt%).

Figure S20. SEM images with a wide field of view to show the uniform morphology of the microcapsules.

Figure S21. Size distribution of the microcapsules.

Figure S22. TGA curves of alkyne/HDI microcapsules (MCs), EPA MCs, shell, pure EPA, and pure alkyne monomer (**1e**) recorded under nitrogen at a heating rate of 10 ^{o}C/min.

Figure S23. Photographs of alkyne/EPA microcapsules with the alkyne content of (A) 0.5 wt%, (B) 1.0 wt%, and (C) 5.0 wt% without catalyst before crushing, mixing with 50 wt% DABCO catalyst before crushing, and mixing with 50 wt% DABCO catalyst after crushing between glass slides for 1 min.

Figure S24. Photographs of alkyne/EPA microcapsules (alkyne content = 5.0 wt%) without catalyst before crushing, mixing with DABCO catalyst before crushing, and mixing with DABCO catalyst after crushing between glass slides for 1 min. The amount of catalyst relative to the dosage of the microcapsules was 50 wt%, 20 wt% and 10 wt% for panel A, B and C, respectively.

Figure S25. Photographs of the mixture of alkyne/EPA microcapsules (alkyne content = 5.0 wt%) and DABCO catalyst before and after crushing between glass slides. The mixture was crushed under the compression of a weight of (A) 200 g, (B) 500 g, and (C) 1 kg. The amount of catalyst relative to the dosage of the microcapsules was 10 wt%.

Figure S26. UV-vis spectra of alkyne/EPA microcapsules (MCs, alkyne content = 5.0 wt%) without catalyst and the mixture of MCs with DABCO catalyst measured at different scan time.

Figure S27. Photographs of PVA films containing (A) no microcapsules or catalyst and (B) 5 wt% DABCO catalyst before and after being scratched with a utility knife for 10 min.

Figure S28. Photographs of PVA films containing different amount of alkyne/EPA microcapsules (alkyne content = 5.0 wt%) and 10 wt% DABCO catalyst: (A) 15 wt% alkyne/EPA microcapsules and 10 wt% DABCO catalyst, (B) 15 wt% alkyne/EPA microcapsules and 5 wt% DABCO catalyst. (C) 10 wt% alkyne/EPA and 10 wt% DABCO catalyst, and (D) 10 wt% alkyne/EPA microcapsules and 5 wt% DABCO catalyst. The photographs were taken before and after the films being scratched with a utility knife for different time.

Figure S29. Photographs of PVA films containing 15 wt% alkyne/EPA microcapsules and 5 wt% DABCO catalyst before and after being impacted and compressed by an iron bar for different time.

EXPERIMENTAL SECTION

General Materials and Instruments

1,4-Diazabicyclo[2.2.2]octane (DABCO), propiolic acid, 1,6-hexanediol, 1,3-propanediol, 2,2-dibutyl-1,3-propanediol, 4,4'-oxydiphenol, and all other chemicals and reagents were purchased from Meryer, J&K Scientific, or Aldrich and used as commercially received unless stated otherwise. Dichloromethane (DCM), tetrahydrofuran (THF), *N,N*-dimethylformamide (DMF), dimethyl sulfoxide (DMSO), and 1,4-dioxane are ultra-dry reagents purchased from J&K Scientific. Poly(vinyl alcohol) 1788 (87.0-89.0% hydrolyzed, mol/mol) was purchased from Aladdin Chemical Ltd. Monomers **1a**–**1e** were synthesized according to the previous reported procedures.[1-3]

Weight- (M_w) and number-average molecular weights (M_n) and polydispersity indices (M_w/M_n) of the polymers were estimated on a Waters e2695 gel permeation chromatography (GPC) system equipped with a refractive index detector and calibrated using a set of monodispersed polystyrene standards. THF was used as the eluent. IR spectra were recorded on a Nicolet 6700 FTIR spectrophotometer (KBr disk). ^{1}H and ^{13}C NMR spectra were measured on a Bruker AVANCE III spectrometer in deuterated chloroform ($CDCl_3$). Chemical shifts were calibrated using $CDCl_3$ as internal reference at δ 7.26 ppm (^{1}H NMR) and δ 77.16 ppm (^{13}C NMR). High-resolution mass spectra (HRMS) were recorded on a GCT premier CAB048 mass spectrometer. UV spectra were measured on a Shimadzu UV-2450 UV/Vis spectrophotometer. Thermogravimetric analysis (TGA) and differential scanning calorimetry (DSC) measurement was carried on a TA TGA 55 and a TA DSC Q200, respectively, under nitrogen at a heating rate of 10 °C/min. Scanning electron microscopy (JEOL-6390) was applied to observe the morphologies of the microcapsules and scratched areas of polymer films. The SEM samples of microcapsule and coatings were coated with a thin layer of gold before observation. The

photographs of microcapsules and polymer films were acquired using a Canon EOS 70D digital camera under ambient room lighting.

Synthesis and Characterization

Characterization of Polymers. *Characterization Data for P**1a***: Brownish red powder; yield: 84% (Table 1, entry 2). M_n = 21800; M_w = 29800; M_w/M_n = 1.4 (GPC for soluble part, polystyrene calibration). FT-IR (KBr disk), v (cm^{-1}): 3005, 2939, 2859, 2220, 1708, 1618, 1463, 1386, 1235, 1169, 958, 744. ^1H NMR (400 MHz, $CDCl_3$), δ (ppm): 7.58 (d, J = 12.0 Hz), 6.78 (d, J = 16.0 Hz), 6.47 (d, J = 16.0 Hz), 5.65 (d, J = 12.0 Hz), 4.23–4.13, 1.70–1.63, 1.41. ^{13}C NMR (100 MHz, $CDCl_3$), δ (ppm): 166.29, 164.89, 157.48, 153.35, 135.48, 121.79, 104.35, 87.18, 81.78, 66.42, 65.34, 64.56, 29.84, 28.65, 28.55, 28.52, 28.39, 25.65, 25.62, 25.58, 25.55.

*Characterization Data for P**1b***: Yellow powder; yield: 84% (Table 1, entry 4). M_n = 700; M_w = 1100; M_w/M_n = 1.6 (GPC for soluble part, polystyrene calibration). FT-IR (KBr disk), v (cm^{-1}): 3081, 2963, 2904, 2221, 1705, 1619, 1461, 1387, 1231, 1168, 1035, 956, 744. ^1H NMR (400 MHz, $CDCl_3$), δ (ppm): 7.58 (d, J = 12.0 Hz), 6.80 (d, J = 16.0 Hz), 6.48 (d, J = 16.0 Hz), 5.67 (d, J = 12.0 Hz), 4.36–4.24, 2.12–2.05. ^{13}C NMR (100 MHz, $CDCl_3$), δ (ppm): 168.09, 164.60, 153.03, 150.76, 135.25, 122.10, 101.65, 85.10, 82.06, 62.77, 61.92, 27.75.

*Characterization Data for P**1c***: Yellow powder; yield: 9% (Table 1, entry 6). M_n = 4200; M_w = 6400; M_w/M_n = 1.5 (GPC for soluble part, polystyrene calibration). FT-IR (KBr disk), v (cm^{-1}): 3058, 2937, 2866, 2231, 1716, 1621, 1466, 1382, 1235, 1124, 1032, 958, 734. ^1H NMR (400 MHz, $CDCl_3$), δ (ppm): 7.56 (d, J = 12.0 Hz), 6.77 (d, J = 16.0 Hz), 6.49 (d, J = 16.0 Hz), 5.66 (d, J = 12.0 Hz), 4.08–3.95, 1.30, 1.21, 0.90. ^{13}C NMR data was not obtained due to the low yield and poor solubility of the polymer.

*Characterization Data for P**1d***: Yellow powder; yield: ~100% (Table 1, entry 9). M_n = 900; M_w = 1600; M_w/M_n = 1.8 (GPC for soluble part, polystyrene calibration). FT-IR (KBr disk), v (cm^{-1}): 3069, 2216, 1723, 1619, 1489, 1223, 1172, 1137, 1093, 1008, 952, 838, 508. ^1H NMR (400 MHz, $CDCl_3$), δ (ppm): 7.81 (d, J = 12.0 Hz), 7.17–7.08, 7.06, 7.04, 7.01, 6.44 (d, J =

16.0 Hz), 5.92 (d, J = 12.0 Hz), 4.08–3.95, 1.30, 1.21, 0.90. ^{13}C NMR data was not obtained due to the poor solubility of the polymer.

*Characterization Data for **P1e***: Brownish red powder; yield: 97% (Table 1, entry 11). FT-IR (KBr disk), v (cm^{-1}): 3016, 2968, 2880, 2231, 1717, 1622, 1465, 1389, 1233, 1163, 1054, 957, 744. ^{1}H NMR (400 MHz, $CDCl_3$), δ (ppm): 6.79 (d, J = 16.0 Hz), 6.81 (d, J = 16.0 Hz), 4.26–4.10, 1.31−1.20, 0.93–0.83. ^{13}C NMR data was not obtained due to the poor solubility of the polymer.

Model Reaction. To a stirred solution of ethyl propiolate (0.51 mL, 5 mmol) in DCM (4 mL) at 0 °C was added the solution of DABCO (5.6 mg, 0.05 mmol) in DCM (1 mL) under air. The reaction immediately turned into brown and was stirred for 1 h. After reaction, the DABCO catalyst was removed by filtration through a pad of silica gel. Then the solvent was evaporated and the crude reaction mixture was purified by silica gel chromatography using hexane/ethyl acetate mixture (95:5, v/v) as eluent. The diethyl (*E*)-hex-2-en-4-ynedioate product (**M1**) was collected as yellow oil in a NMR yield: 93.8%. The byproduct diethyl 3,3'-oxy(2*E*,2'*E*)-diacrylate (**M2**) was obtained as white solid in an NMR yield of 6.2%. The structural characterization results are summarized as follows.

*Characterization Data for **M1**:* ^{1}H NMR (400 MHz, $CDCl_3$), δ (ppm): 6.70 (d, J = 16.0 Hz, 1H), 6.39 (d, J = 16.0 Hz, 1H), 4.22–4.15 (m, 4H), 1.26–1.21 (m, 6H). ^{13}C NMR (100 MHz, $CDCl_3$), δ (ppm): 164.12, 152.51, 135.05, 121.10, 86.70, 80.93, 61.96, 60.87, 13.72, 13.56. FT-IR (KBr disk), v (cm^{-1}): 3038, 2981, 2238, 1710, 1618, 1459, 1368, 1243, 1175, 1020, 859. HRMS (CI, CH_4): m/z [M + H]$^+$ calcd for $C_{10}H_{12}O_4$, 196.0736; found, 197.0810.

*Characterization Data for **M2**:* ^{1}H NMR (400 MHz, $CDCl_3$), δ (ppm): 7.57 (d, J = 12.0 Hz, 2H), 5.64 (d, J = 12.0 Hz, 2H), 4.19 (q, J = 7.0 Hz, 4H), 1.28 (t, J = 7.0 Hz, 6H). ^{13}C NMR (100 MHz, $CDCl_3$), δ (ppm): 166.19, 157.37, 104.43, 60.64, 14.37. FT-IR (KBr disk), v (cm^{-1}): 3087,

2989, 2925, 1710, 1608, 1474, 1368, 1330, 1195, 1120, 967, 864, 846, 735, 585. HRMS (CI, CH_4): *m*/*z* $[M + H]^+$ calcd for $C_{10}H_{14}O_5$, 214.0841; found, 215.0912.

Supporting References

[1] Si, H.; Wang, K. J.; Song, B.; Qin, A. J.; Tang, B. Z. *Polym. Chem.* **2020**, *11*, 2568-2575.

[2] Li, H. K.; Wang, J.; Sun, J. Z.; Hu, R. R.; Qin, A. J.; Tang, B. Z. *Polym. Chem.* **2012**, *3*, 1075-1083.

[3] Wang, X.; Hu, R. R.; Zhao, Z. J.; Qin, A. J.; Tang, B. Z. *Sci. China Chem.* **2016**, *59*, 1554-1560.

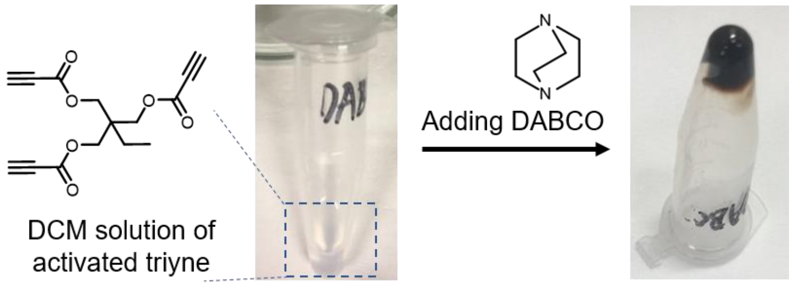

Figure S1. Photographs showing the chromogenic phenomenon upon adding DABCO crystals into the dichloromethane (DCM) solution of activated triyne.

Table S1. Preliminary Trials on the Polymerization of **1a**[a]

entry	condition	yield (%)	M_w[b]	M_w/M_n[b]	S[c]
1	adding DABCO solution to **1a** solution	81	34 100	1.9	Δ
2	adding **1a** solution to DABCO solution	41	18 500	1.3	Δ
3	adding **1a** solution to DABCO powder	61	18 200	1.4	Δ
4	adding DABCO solution to **1a** powder	70	17 100	1.4	Δ
5	mixing the powder of DABCO and **1a**	73	18 000	1.4	Δ
6[d]	mixing the powder of DABCO and **1a**	96	/[f]		×
7[e]	mixing the powder of DABCO and **1a**	96	/[f]		×

[a] Unless otherwise noted, the polymerizations were carried out at 0 ºC in anhydrous DCM in air for 1 h. [**1a**] = 0.2 M, [DABCO] = 10 mol% [**1a**]. [b] Determined by GPC in THF on the basis of a linear polystyrene calibration. [c] Solubility (S) tested in common organic solvents, such as DCM, chloroform, and THF: Δ = partially soluble, × = insoluble. The GPC results are for the soluble part. [d] [DABCO] = 50 mol%. [e] Carried out under room temperature. [f] Not detected.

Table S2. Effect of Solvent on the Polymerization of **1a**[a]

entry	solvent	yield (%)	M_w[b]	M_w/M_n[b]	S[c]
1[d]	DCM	81	34 100	1.9	Δ
2	DCM/water = 1:1 (v/v)	trace			Δ
3	DCM/water = 3:2 (v/v)	trace			Δ
4	THF	32	6 800	1.3	Δ
5	THF/water = 1:1 (v/v)	3	2 800	1.1	Δ
6	DMF	59	2 900	1.1	Δ
7	DMSO	65	/[e]		×
8	1,4-dioxane	gel, ~100	14 600	1.5	Δ
9	hexyl acetate	60	9 000	1.3	Δ
10	ethyl phenylacetate	5	2 800	1.1	Δ
11	MeCN	~100	/[e]		×
12	toluene	27	2 800	1.1	Δ

[a] Unless otherwise noted, the polymerizations were carried out at 0 °C under air for 1 h by adding DABCO solution to **1a** solution. [**1a**] = 0.2 M, [DABCO] = 10 mol% [**1a**]. [b] Determined by GPC in THF on the basis of a linear polystyrene calibration. [c] Solubility (S) tested in common organic solvents, such as DCM, chloroform, and THF: Δ = partially soluble, × = insoluble. The GPC results are for the soluble part. [d] Data taken from Table S1, entry 2. [e] Not detected.

Table S3. Time Course of the Polymerization of **1a**[a]

entry	time	yield (%)	M_w[b]	M_w/M_n[b]	S[c]
1	5 min	23	/[e]		Δ
2	15 min	63	/[e]		Δ
3	30 min	74	1 000	2.5	Δ
4[d]	1 h	84	29 800	1.4	Δ

[a] Unless otherwise noted, the polymerizations were carried out at 0 °C in anhydrous DCM under air by adding DABCO solution to **1a** solution. [**1a**] = 0.2 M, [DABCO] = 15 mol% [**1a**]. [b] Determined by GPC in THF on the basis of a linear polystyrene calibration. [c] Solubility (S) tested in common organic solvents, such as DCM, chloroform, and THF: Δ = partially soluble. The GPC results are for the soluble part. [d] Data taken from Table S3, entry 1. [e] Not detected.

Table S4. Effect of Catalyst Type on the Polymerization of **1a**[a]

entry	catalyst	yield (%)	M_w[b]	M_w/M_n[b]	Color[c]
1	DABCO	84	29 800	1.4	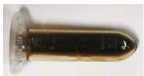
2	DMAP	brownish red solution; undesired and unidentified product			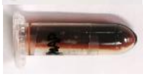
3	NEt_3	deep brownish red solution; no polymeric product (@ *r.t.* + *1 equiv.* NEt_3: deep brownish red gel)			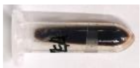
4	pyridine	colorless solution; no polymeric product (@ *r.t.* + *7 equiv. pyridine*: brownish red gel)			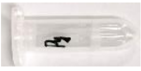
5	4-picoline	light yellow solution; no polymeric product (@ *r.t.* + *2 equiv. 4-picoline*: brownish red gel)			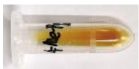
6	DBU	yellow solution; no polymeric product (@ *r.t.* + *10 equiv. DBU*: yellow solution; no polymeric product)			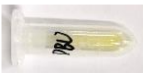
7	NaOH, Na_2CO_3, CH_3COONa	colorless solution with insoluble catalysts; no polymeric product			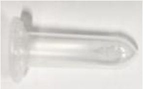

[a] Unless otherwise noted, the polymerizations were carried out at 0 °C in anhydrous DCM under air for 1 h by adding catalyst to **1a** solution. [**1a**] = 0.2 M, [catalyst] = 15 mol% [**1a**]. The contents in parentheses are the polymerization results obtained under the noted conditions. [b] Determined by GPC in THF on the basis of a linear polystyrene calibration. The GPC results are for the soluble part. [c] Photographs showing the appearance color of the reaction mixture after reacting for 1 h. Abbreviation: DMAP = 4-dimethylaminopyridine; DBU = 1,8-diazabicyclo[5.4.0]undec-7-ene; r.t. = room temperature.

Table S5. Polymerization results of monomer **1a–1e**[a]

entry	monomer	[monomer] (M)	yield (%)	M_w[b]	M_w/M_n[b]	S[c]
1	**1a**	0.2	84	29 800	1.4	Δ
2	**1b**	0.2	49	1 900	1.1	Δ
3[d]	**1b**	0.2	84	1 100	1.6	Δ
4	**1c**	0.2	trace			
5	**1c**	0.4	9	6 400	1.5	Δ
6	**1d**	0.2 or 0.1		gelled within seconds		
7	**1d**	0.05		gelled within 2 min		
8[e]	**1d**	0.05	~100	1 600	1.8	Δ
9	**1e**	0.2 or 0.1		gelled within seconds		
10	**1e**	0.05	97	/[f]		×

[a] Unless otherwise noted, the polymerizations were carried out at 0 °C in anhydrous DCM in the presence of 15 mol% DABCO in air for 1 h. [b] Determined by GPC in THF on the basis of a linear polystyrene calibration. [c] Solubility (S) tested in common organic solvents, such as DCM, chloroform, and THF: Δ = partially soluble, × = insoluble. The GPC results are for the soluble part. [d] Polymerization results obtained at 2 h. [e] [DABCO] = 5 mol% [**1d**]. [f] Not detected.

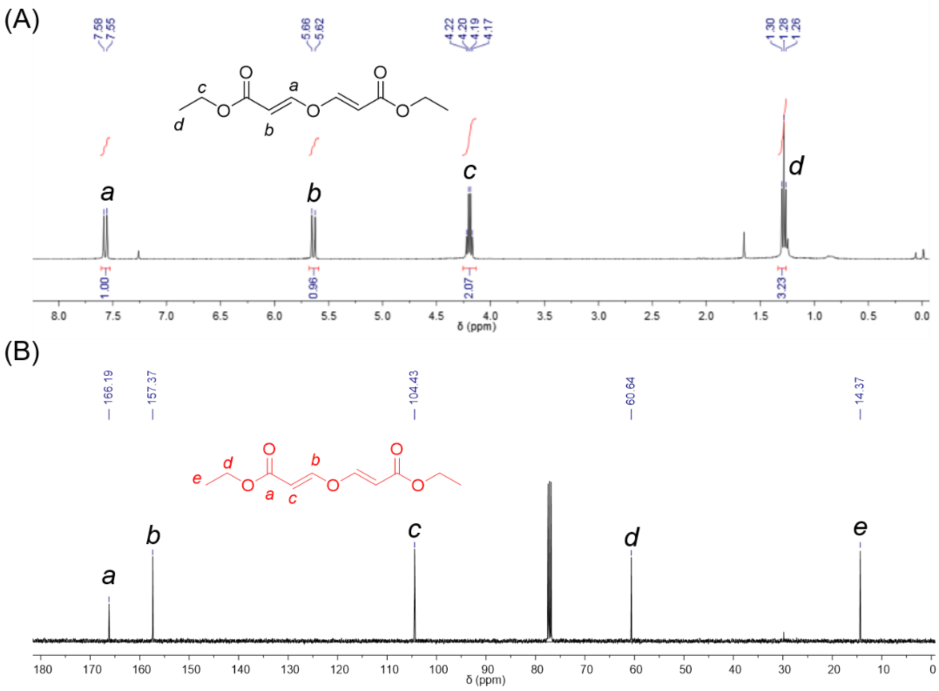

Figure S2. (A) ^{1}H NMR spectrum and (B) ^{13}C NMR spectrum of model compound **M2** in chloroform-*d*.

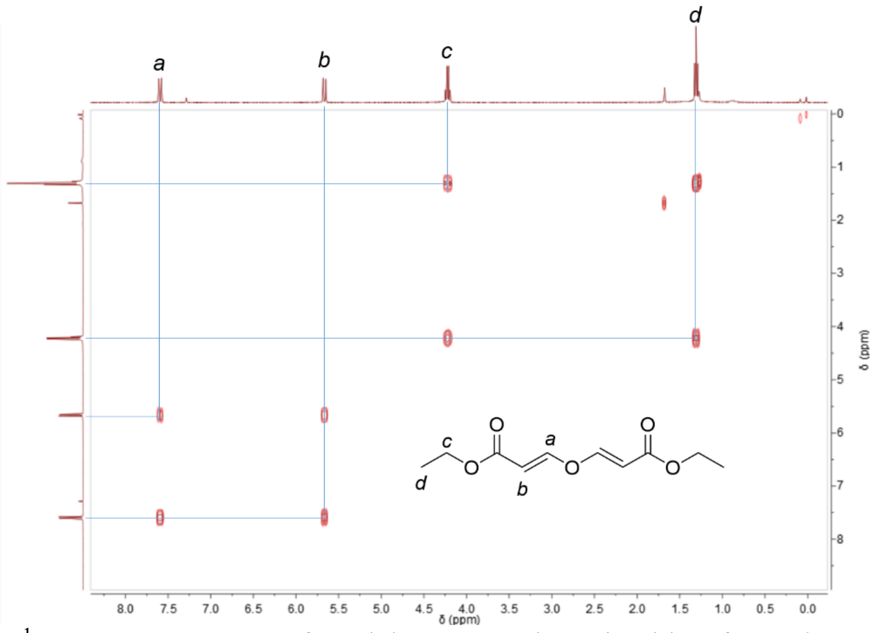

Figure S3. ^{1}H-^{1}H COSY spectrum of model compound **M2** in chloroform-*d*.

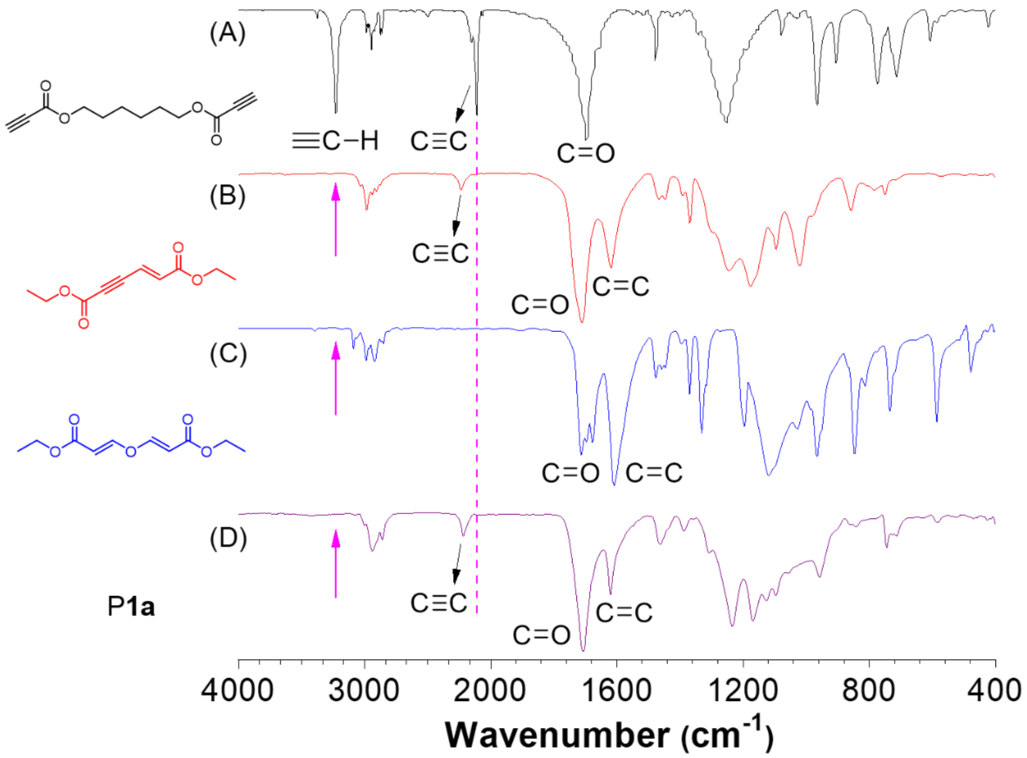

Figure S4. FT-IR spectra of (A) monomer **1a**, (B) model compound **M1**, (C) model compound **M2**, and (D) polymer P**1a**.

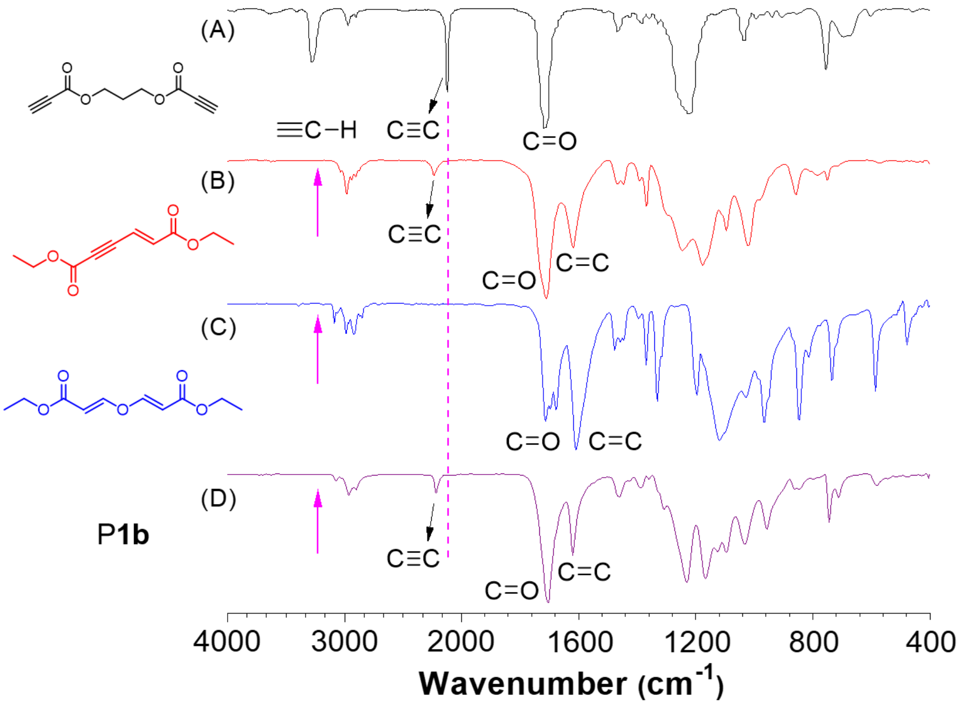

Figure S5. FT-IR spectra of (A) monomer **1b**, (B) model compound **M1**, (C) model compound **M2**, and (D) polymer P**1b**.

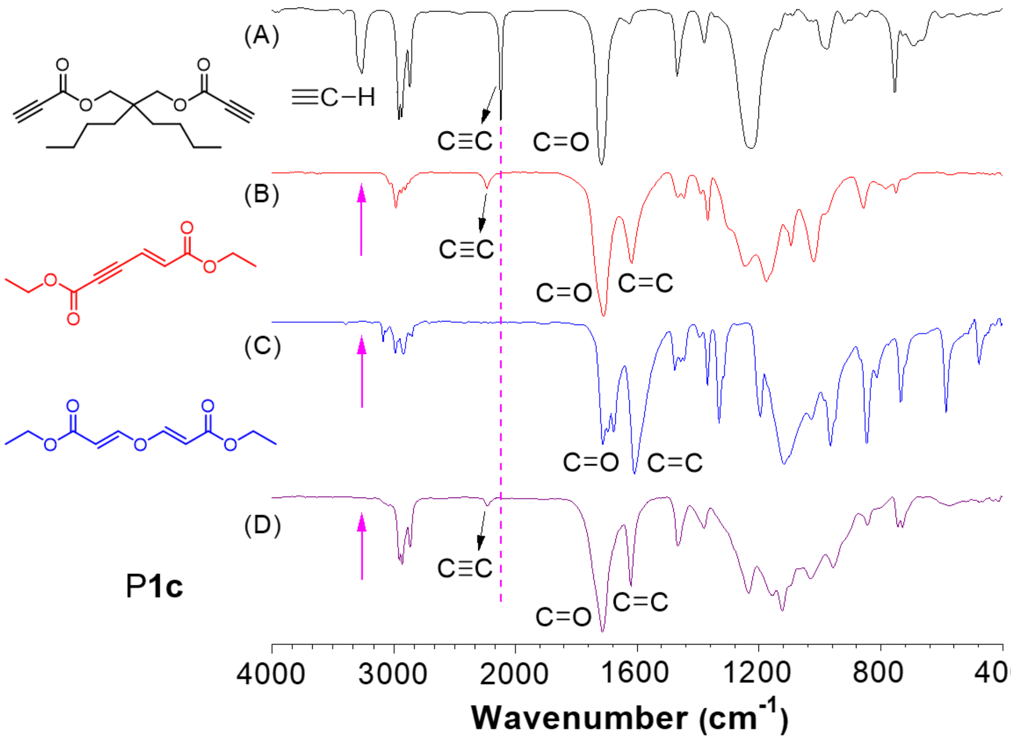

Figure S6. FT-IR spectra of (A) monomer **1c**, (B) model compound **M1**, (C) model compound **M2**, and (D) polymer P**1c**.

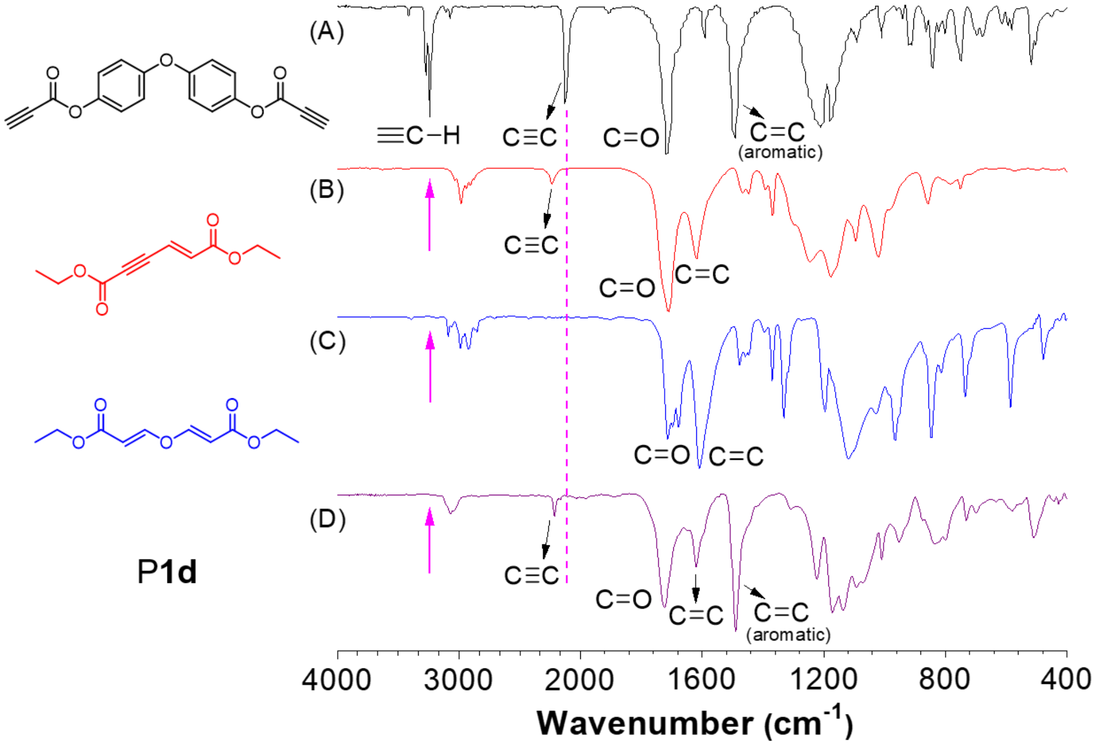

Figure S7. FT-IR spectra of (A) monomer **1d**, (B) model compound **M1**, (C) model compound **M2**, and (D) polymer P**1d**.

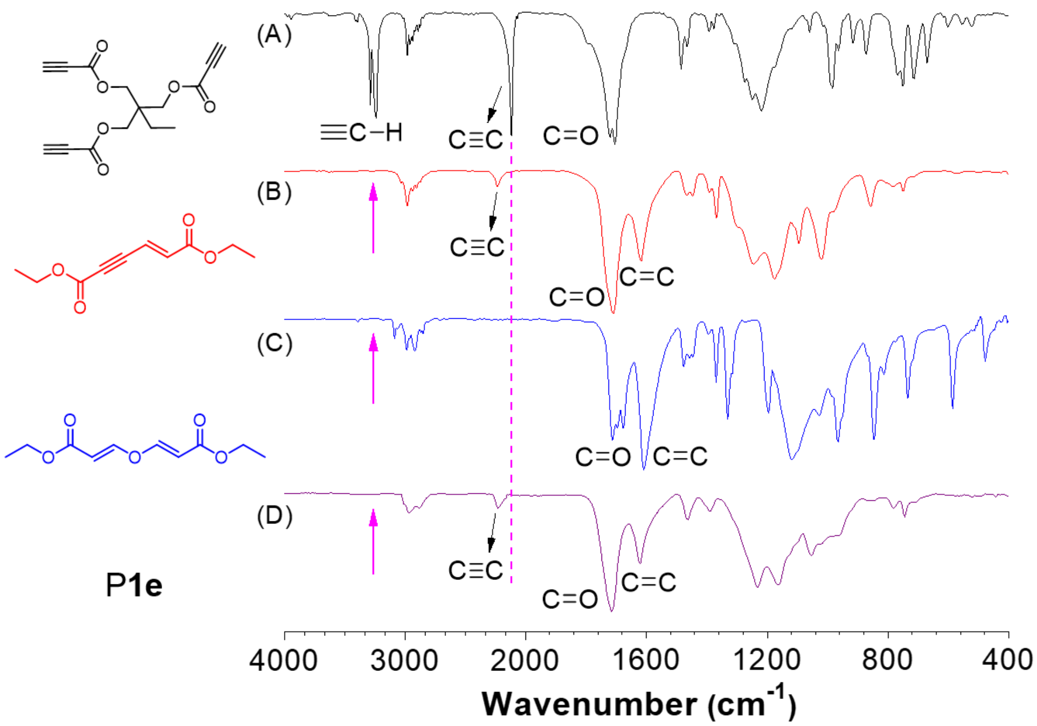

Figure S8. FT-IR spectra of (A) monomer **1e**, (B) model compound **M1**, (C) model compound **M2**, and (D) polymer P**1e**.

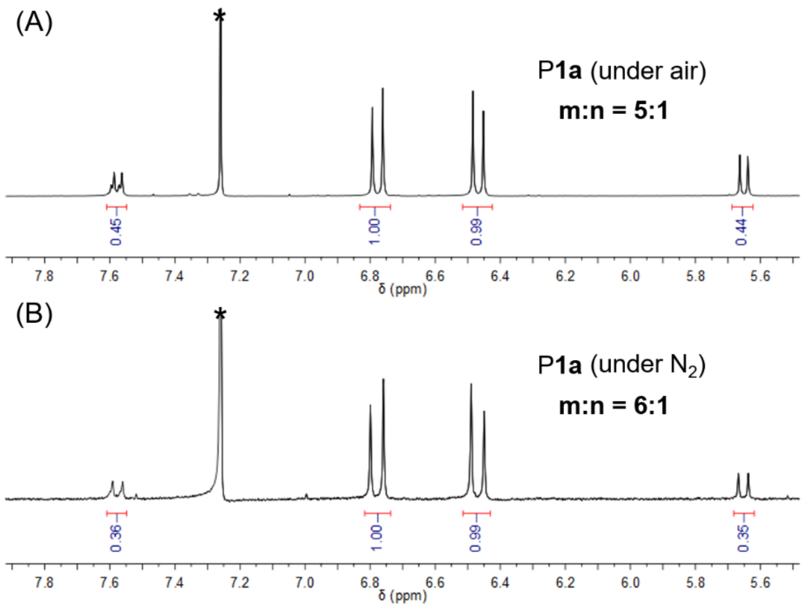

Figure S9. ^{1}H NMR spectra of (A) polymer P**1a** obtained in air and (B) polymer P**1a** obtained under nitrogen. The ^{1}H NMR spectra were measured in chloroform-*d*. The solvent peaks are marked with asterisks.

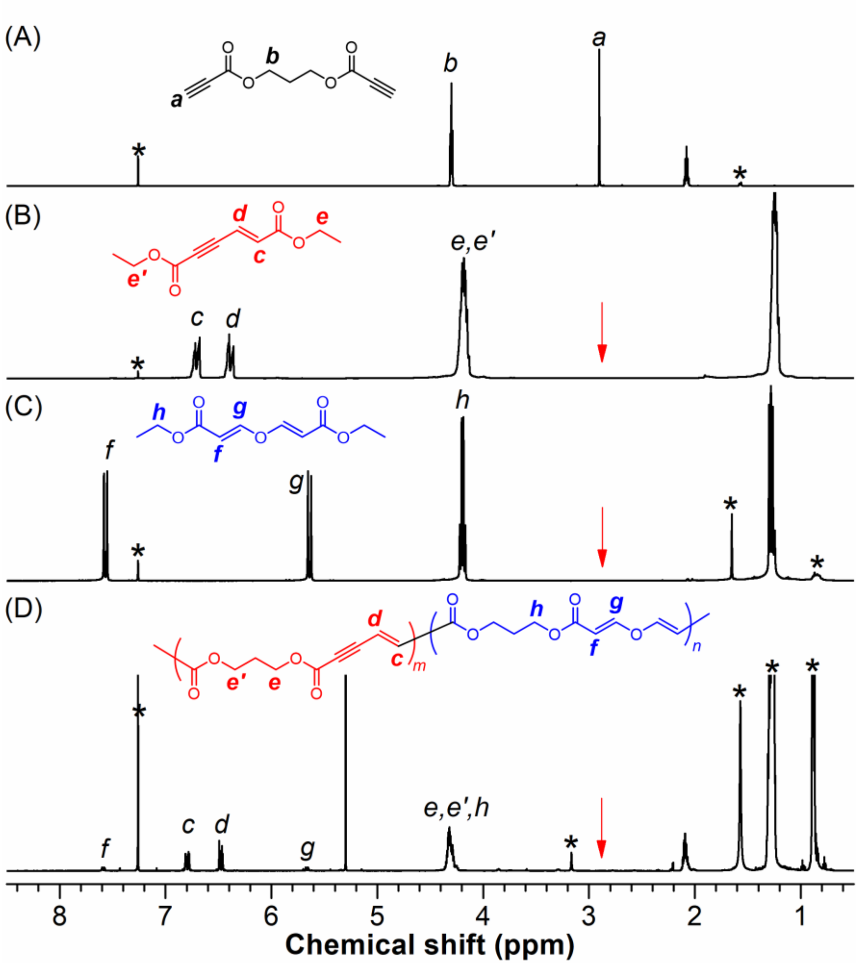

Figure S10. ^{1}H NMR spectra of (A) monomer **1b**, (B) model compound **M1**, (C) model compound **M2**, and (D) polymer P**1b** in chloroform-*d*. The solvent peaks are marked with asterisks.

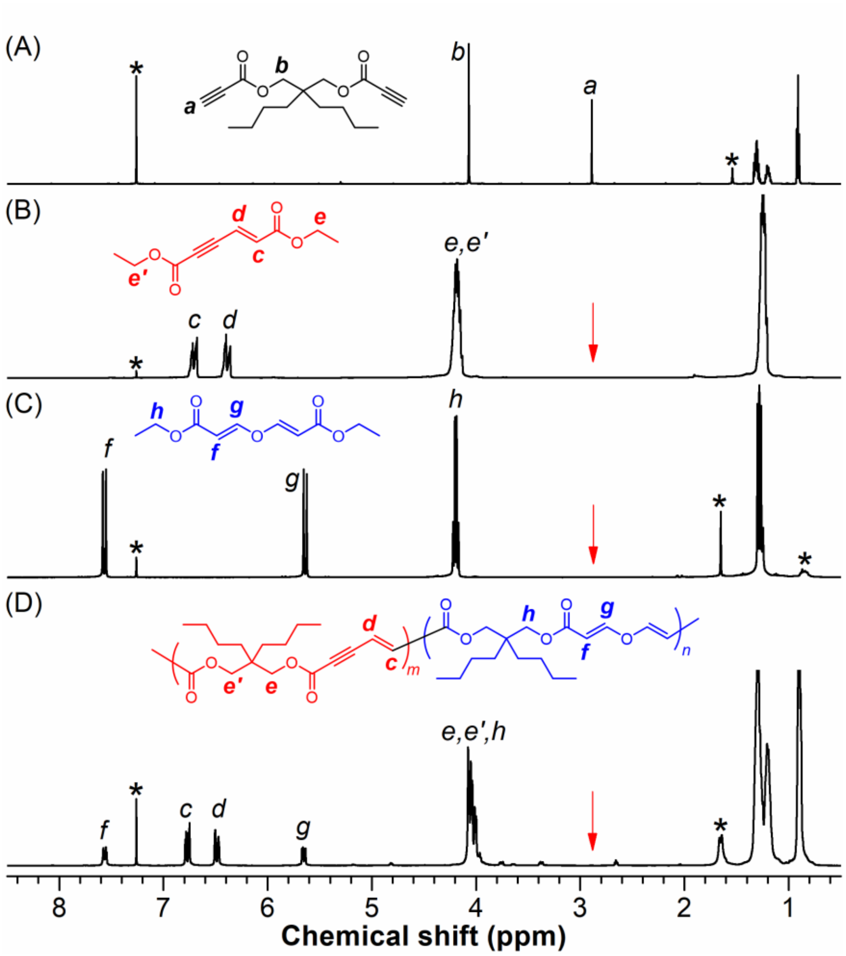

Figure S11. ^{1}H NMR spectra of (A) monomer **1c**, (B) model compound **M1**, (C) model compound **M2**, and (D) polymer P**1c** in chloroform-*d*. The solvent peaks are marked with asterisks.

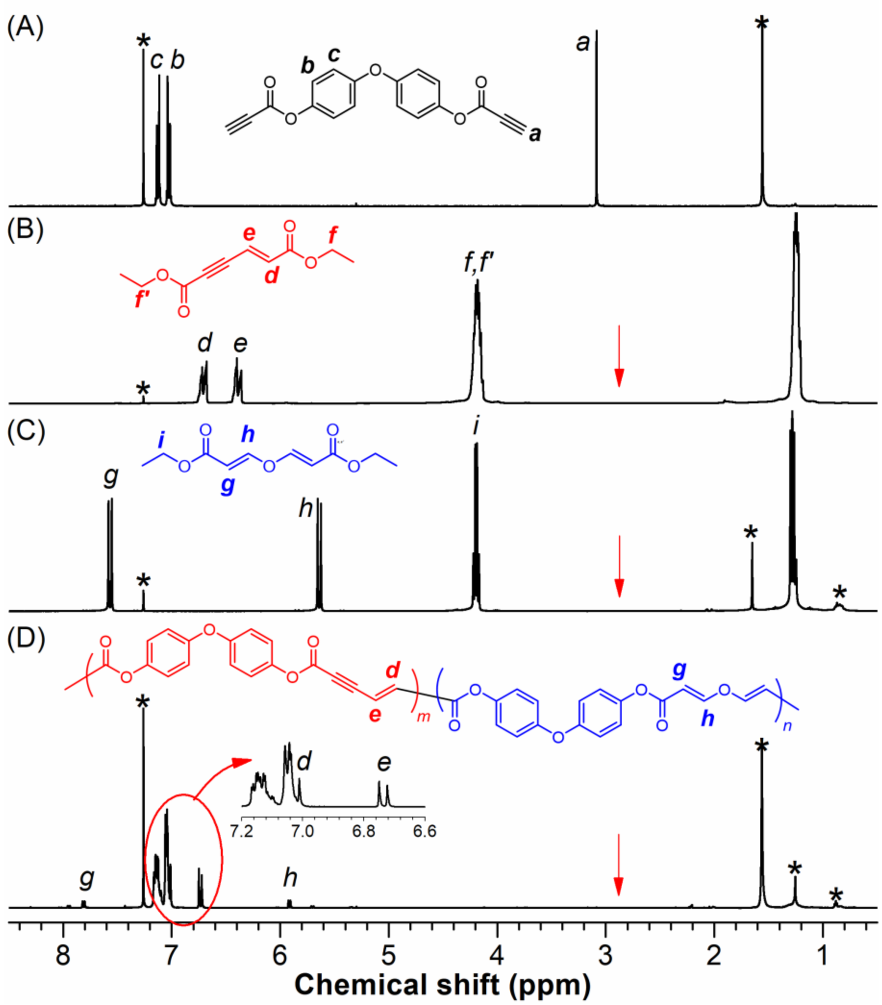

Figure S12. ^{1}H NMR spectra of (A) monomer **1d**, (B) model compound **M1**, (C) model compound **M2**, and (D) polymer P**1d** in chloroform-*d*. The solvent peaks are marked with asterisks.

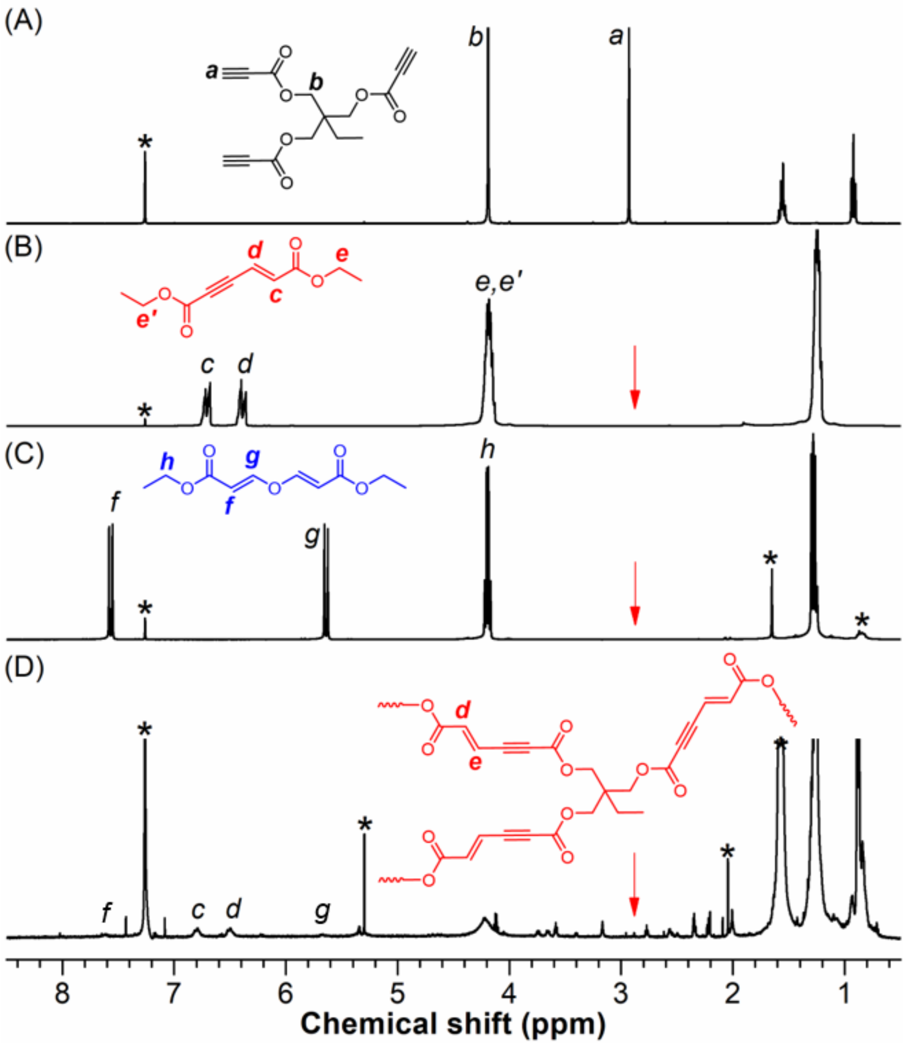

Figure S13. ^{1}H NMR spectra of (A) monomer **1e**, (B) model compound **M1**, (C) model compound **M2**, and (D) polymer P**1e** in chloroform-*d*. The solvent peaks are marked with asterisks.

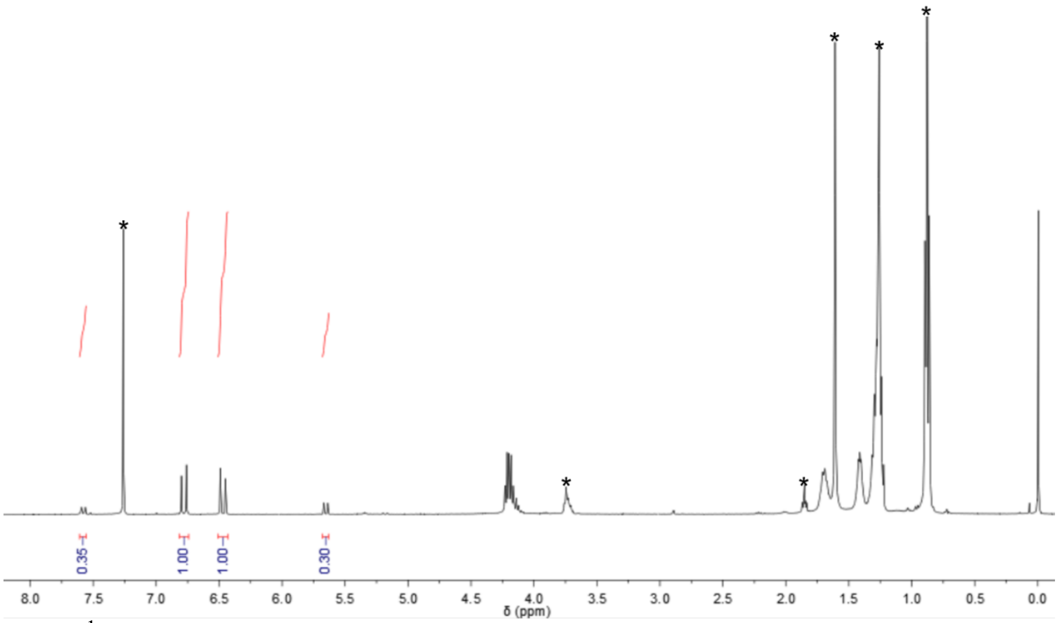

Figure S14. ^{1}H NMR spectrum of polymer P**1a** in chloroform-*d*. The sample was obtained from the polymerization in THF/water mixture (v/v = 1:1). The solvent peaks are marked with asterisks.

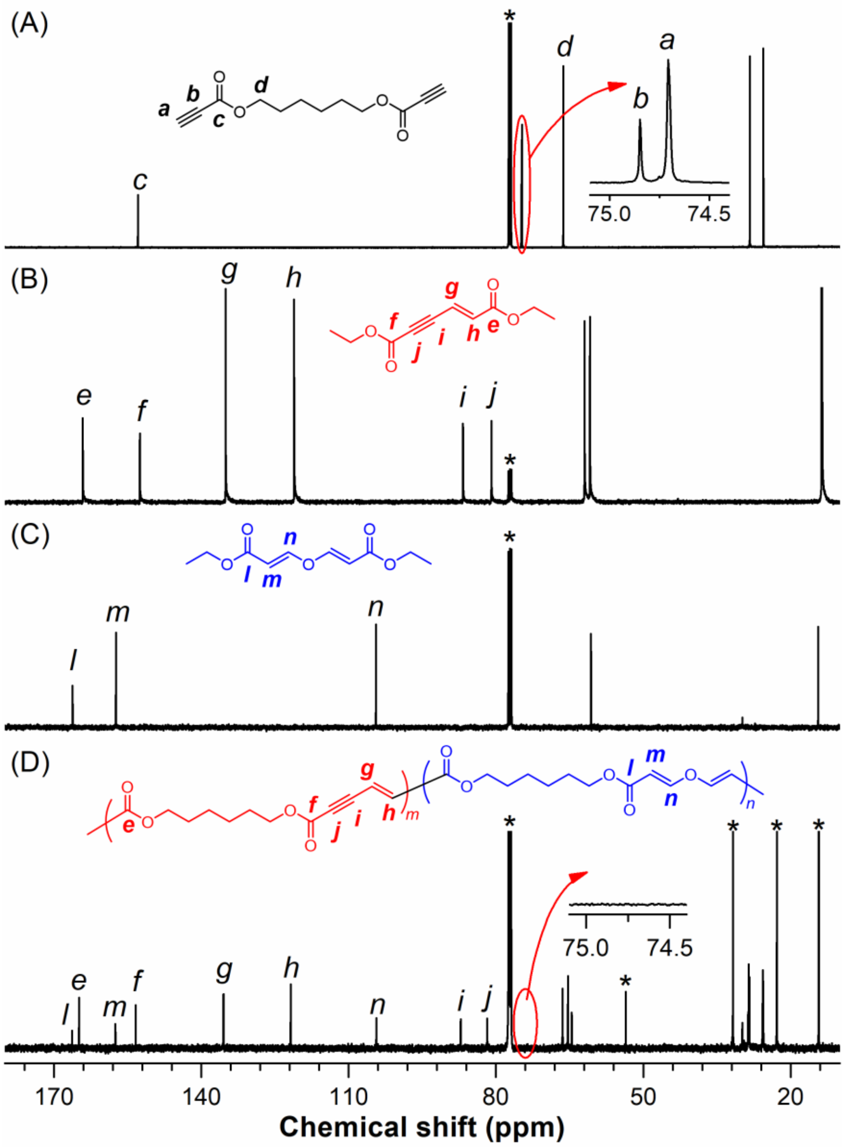

Figure S15. ^{13}C NMR spectra of (A) monomer **1a**, (B) model compound **M1**, (C) model compound **M2**, and (D) polymer P**1a** in chloroform-*d*. The solvent peaks are marked with asterisks.

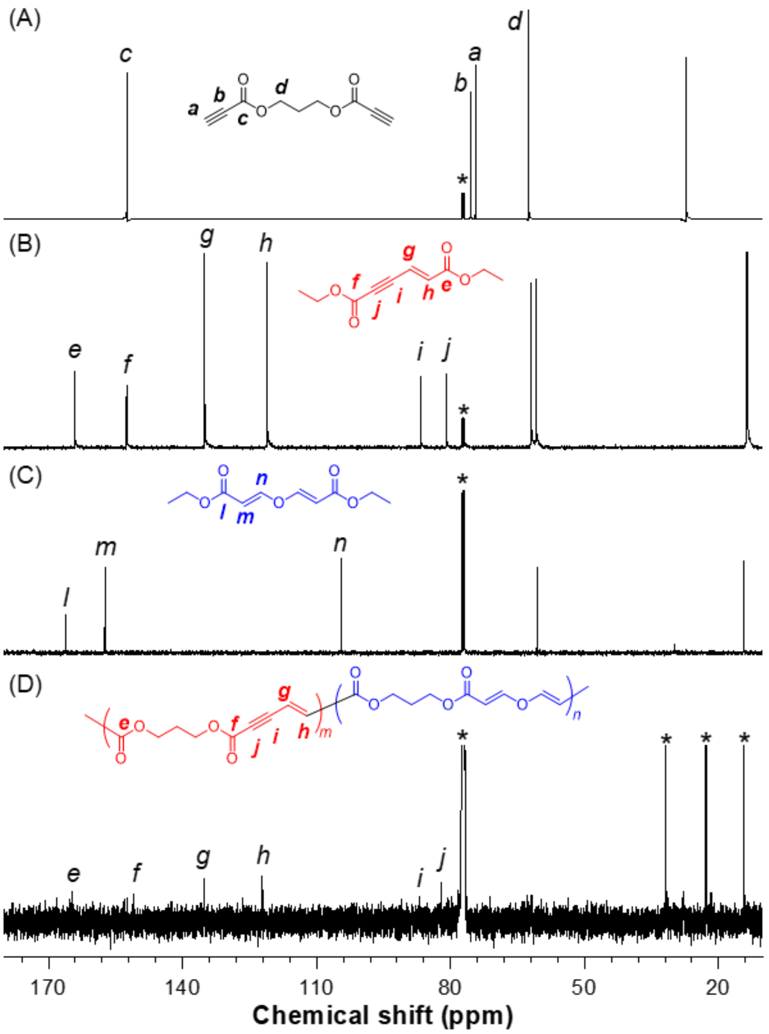

Figure S16. ^{13}C NMR spectra of (A) monomer **1b**, (B) model compound **M1**, (C) model compound **M2**, and (D) polymer **P1b** in chloroform-*d*. The solvent peaks are marked with asterisks.

Scheme S1. Proposed mechanism for the formation of poly(1,3-enyne).

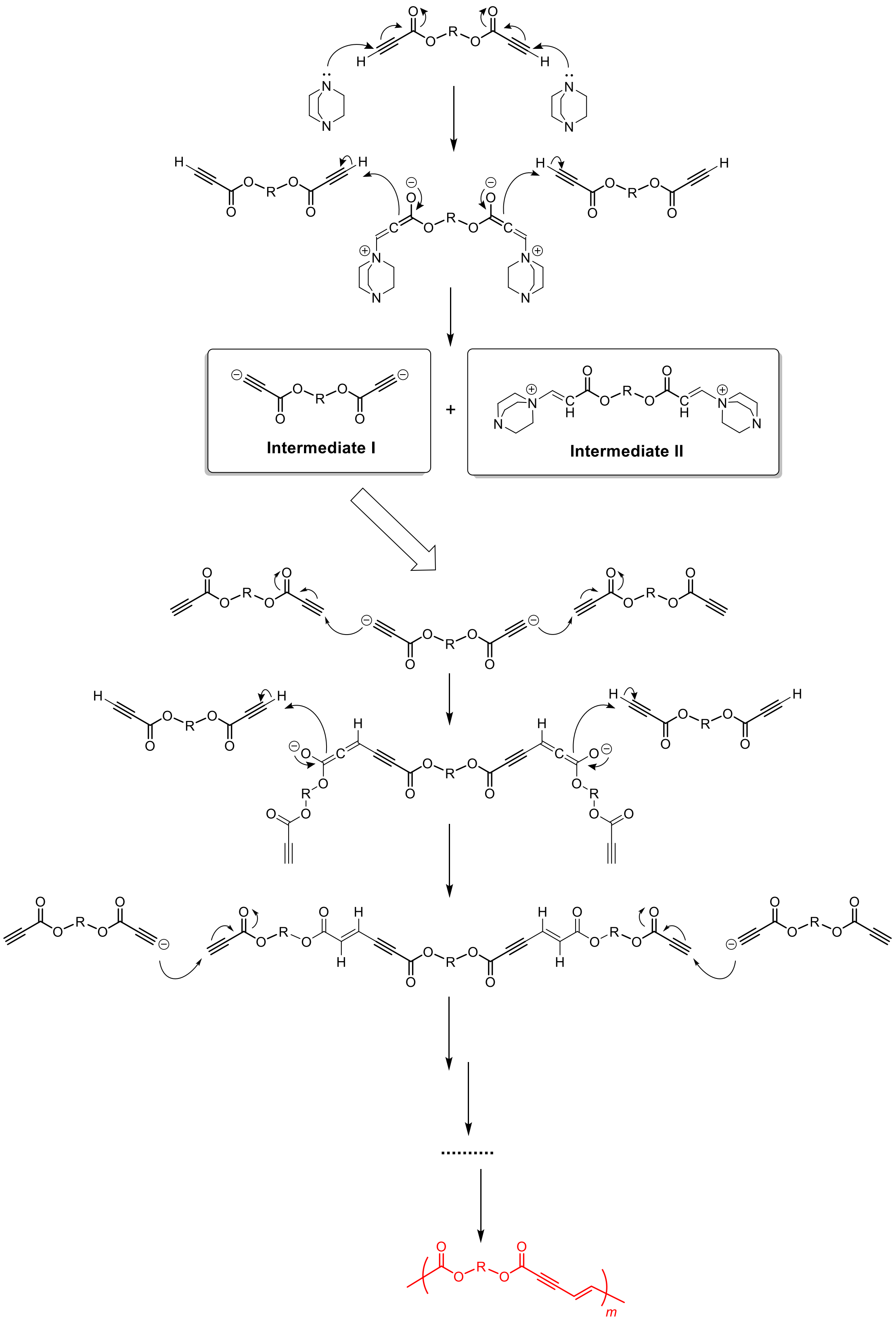

Scheme S2. Proposed mechanism for the formation of copolymers.

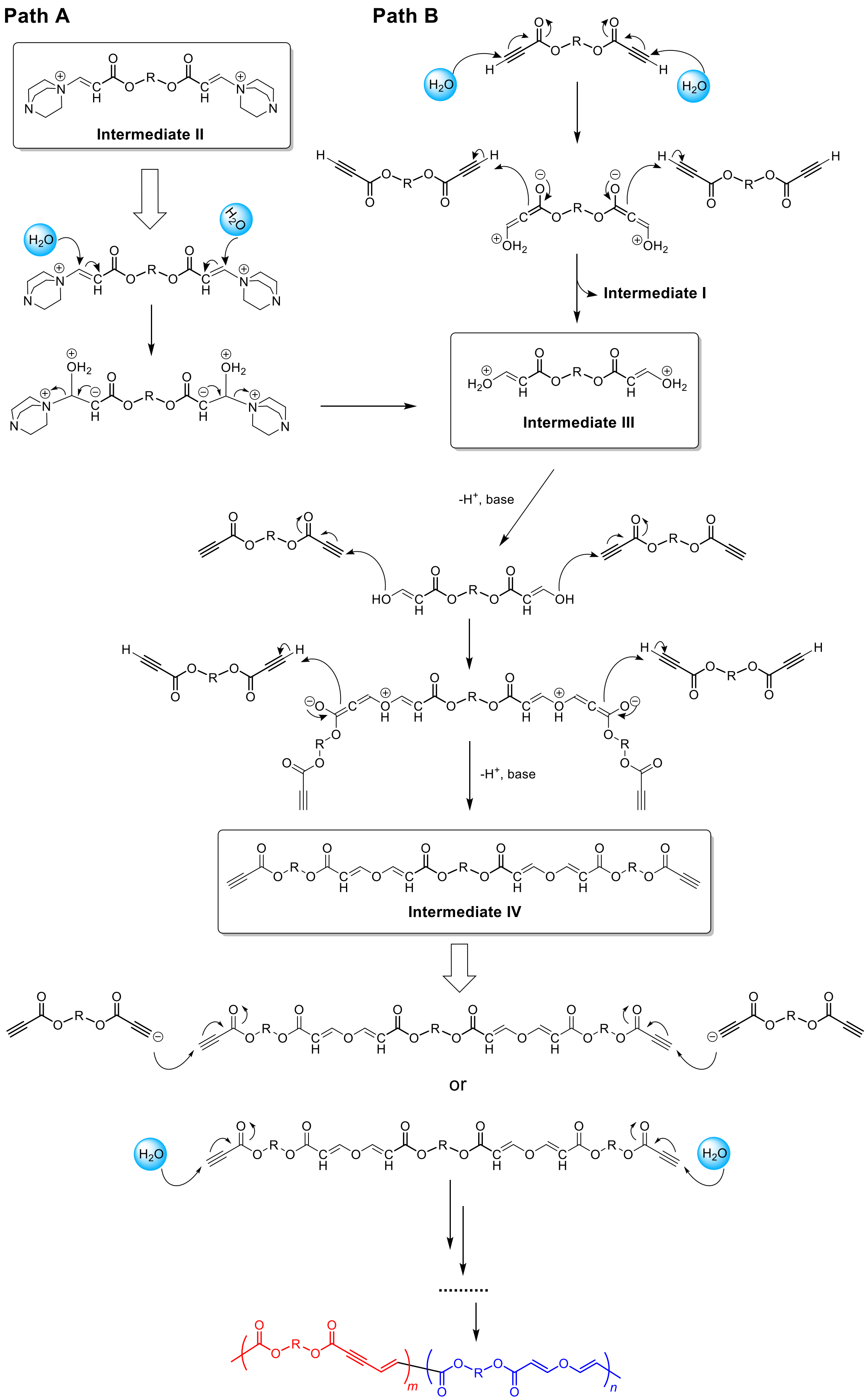

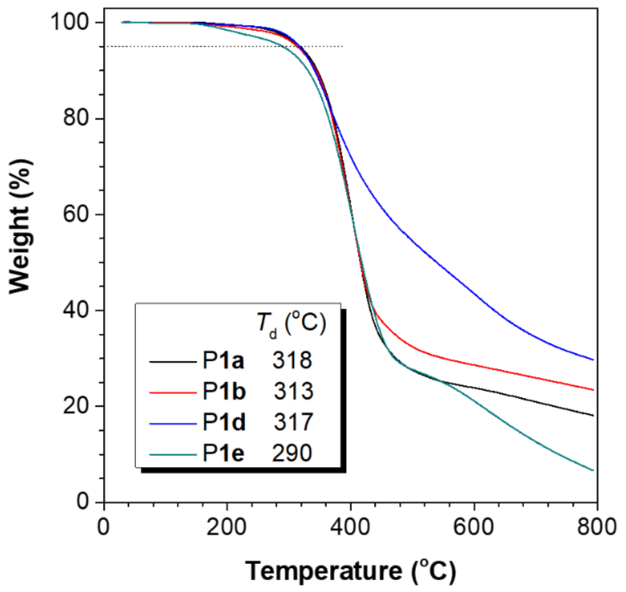

Figure S17. TGA thermograms of polymers recorded under nitrogen at a heating rate of 10 °C/min.

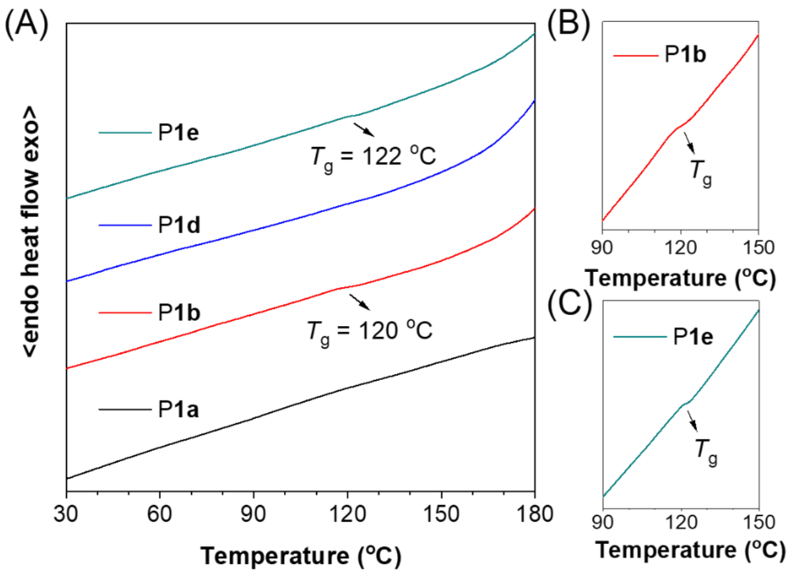

Figure S18. (A) DSC thermograms of polymers and the amplified DSC curve of (B) P**1b** and (C) P**1e** recorded under nitrogen during the second heating cycle at a heating rate of 10 °C/min.

Table S6. Appearance of Polymers P**1a**–P**1e**[a]

entry	monomer structure	photos of polymeric product
1	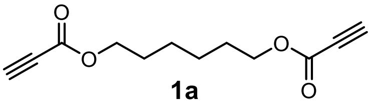 **1a**	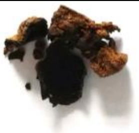
2	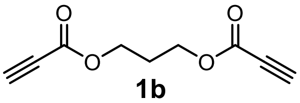 **1b**	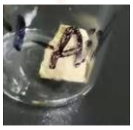
3[b]	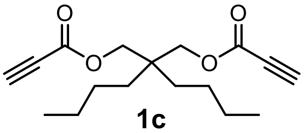 **1c**	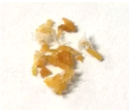
4	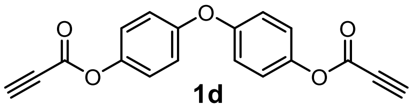 **1d**	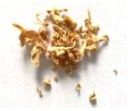
5	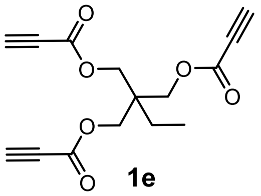 **1e**	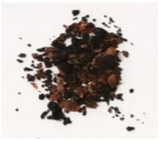

[a] The polymeric products were carried out at 0 °C in anhydrous DCM under air for 1 h by adding DABCO catalyst to the solution of monomer. [monomer] = 0.2 M, [DABCO] = 15 mol% [monomer]. [b] The polymeric product was obtained at a monomer concentration of 0.4 M.

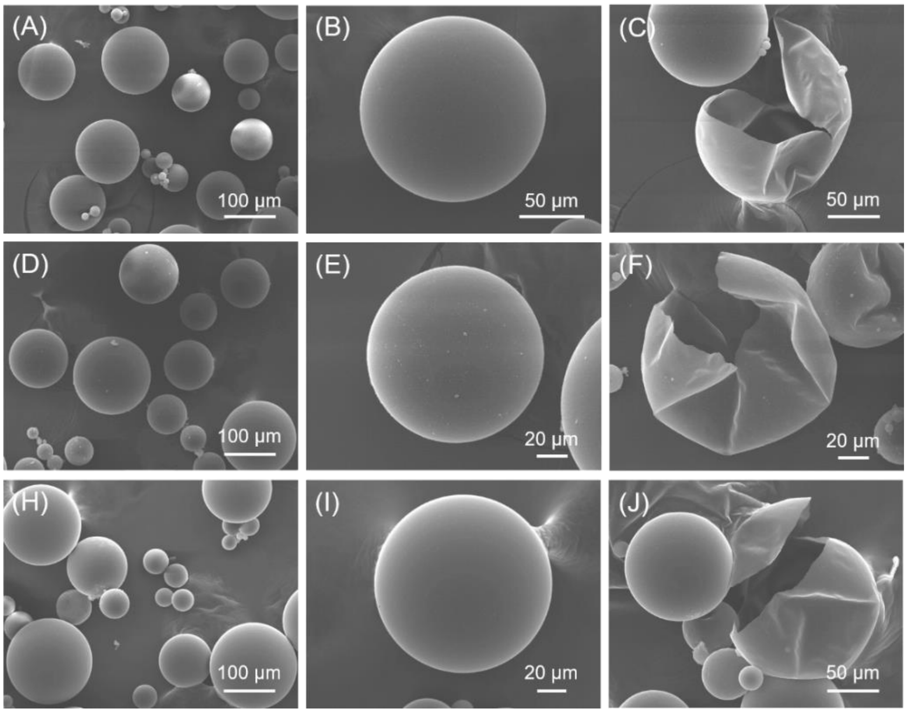

Figure S19. SEM images of intact and ruptured microcapsules containing (A-C) pure EPA solvent, (D-F) alkyne/EPA solution (0.5 wt%), and (H-J) alkyne/EPA solution (5.0 wt%).

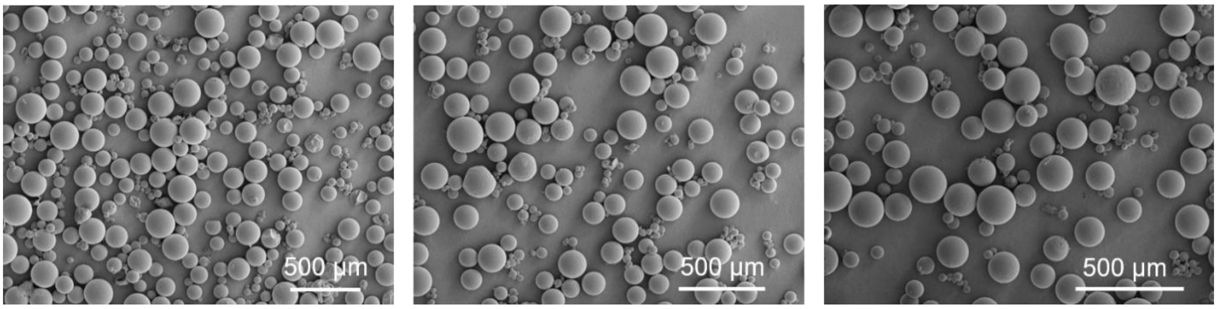

Figure S20. SEM images with a wide field of view to show the uniform morphology of the microcapsules.

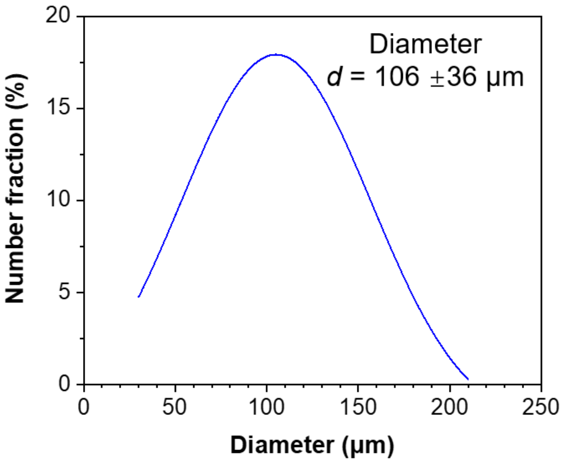

Figure S21. Size distribution of the microcapsules (sample size n = 200). Inset: quantitative data for the size distribution of the microcapsules shown as mean ± standard deviation (S.D.).

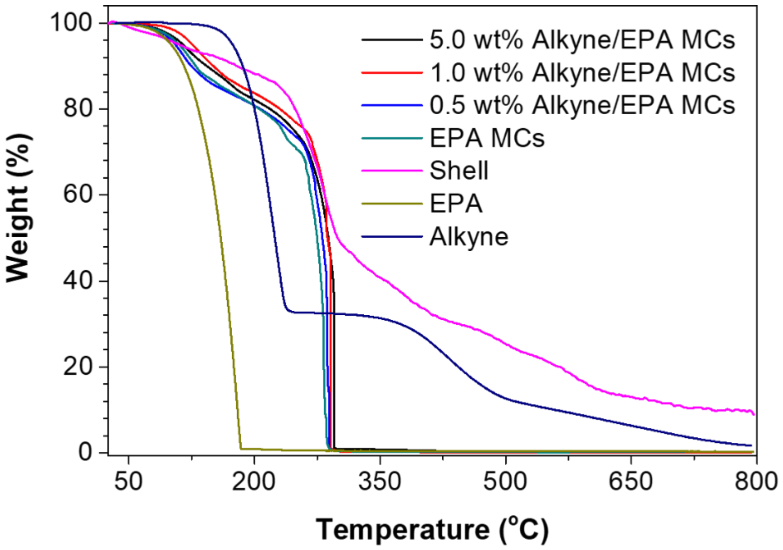

Figure S22. TGA curves of alkyne/HDI microcapsules (MCs), EPA MCs, shell, pure EPA, and pure alkyne monomer (**1e**) recorded under nitrogen at a heating rate of 10 ºC/min.

Effect of alkyne content of microcapsules on damage indication

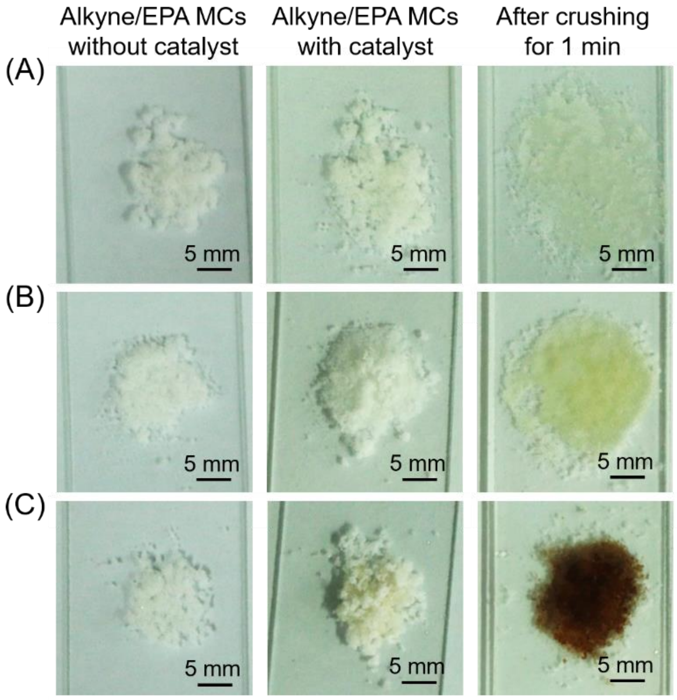

Figure S23. Photographs of alkyne/EPA microcapsules (MCs) with the alkyne content of (A) 0.5 wt%, (B) 1.0 wt%, and (C) 5.0 wt% without catalyst before crushing, mixing with 50 wt% DABCO catalyst before crushing, and mixing with 50 wt% DABCO catalyst after crushing between glass slides for 1 min. The amount of catalyst was relative to the dosage of the microcapsules.

Effect of catalyst dosage on damage indication

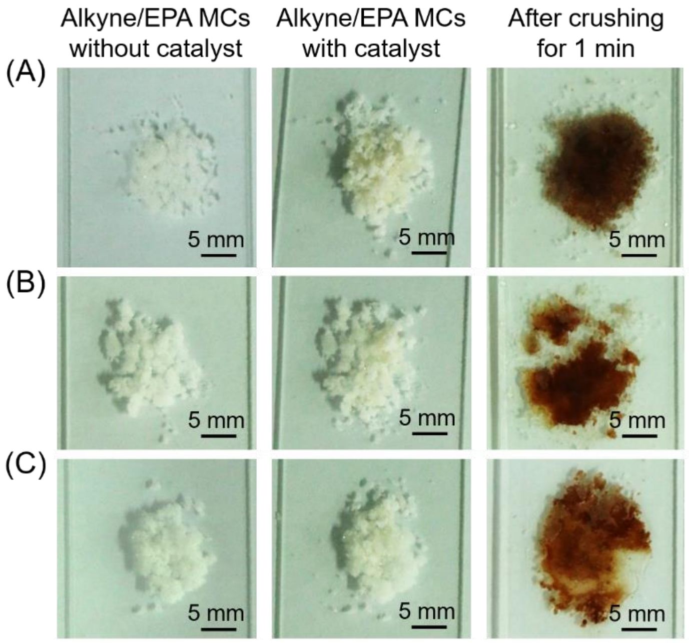

Figure S24. Photographs of alkyne/EPA microcapsules (MCs, alkyne content = 5.0 wt%) without catalyst before crushing, mixing with DABCO catalyst before crushing, and mixing with DABCO catalyst after crushing between glass slides for 1 min. The amount of catalyst relative to the dosage of the microcapsules was 50 wt%, 20 wt% and 10 wt% for panel A, B and C, respectively.

Quantative crushing experiments under different pressure

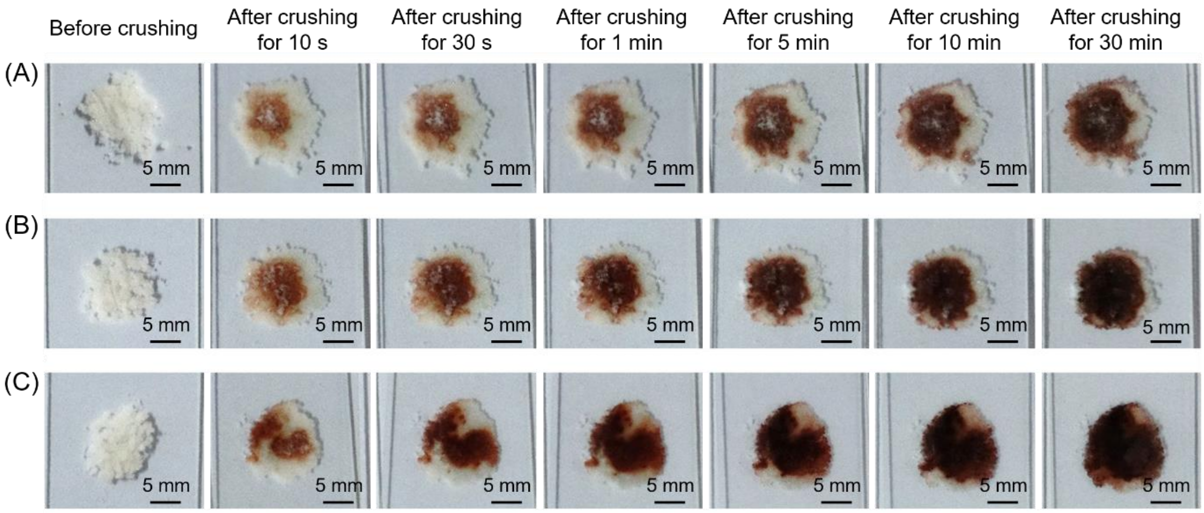

Figure S25. Photographs of the mixture of alkyne/EPA microcapsules (alkyne content = 5.0 wt%) and DABCO catalyst before and after crushing between two glass slides under different intensity of pressure. The mixture was crushed under the compression of a weight of (A) 200 g, (B) 500 g, and (C) 1 kg. The amount of catalyst relative to the dosage of the microcapsules was 10 wt%. All these samples share a similar stress area of about 1.5 cm^2.

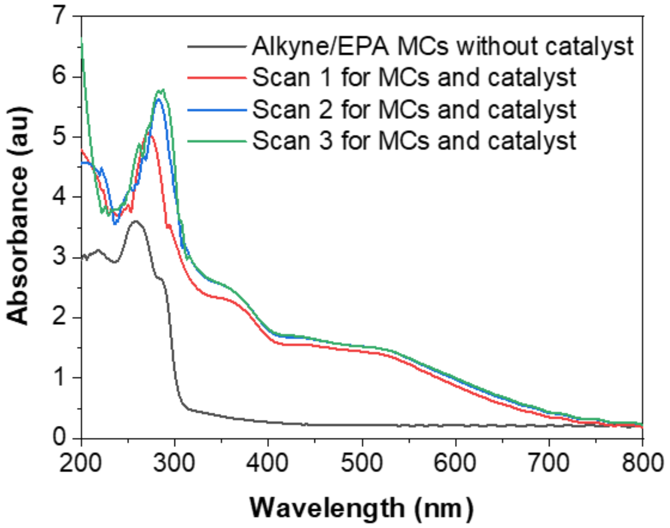

Figure S26. UV-vis spectra of alkyne/EPA microcapsules (MCs, alkyne content = 5.0 wt%) without catalyst and the mixture of MCs with DABCO catalyst measured at different scan time. The spectra were measure by fixing and crushing the MCs or the mixture via the compression between two quartz plates. Each scan takes about 1 min.

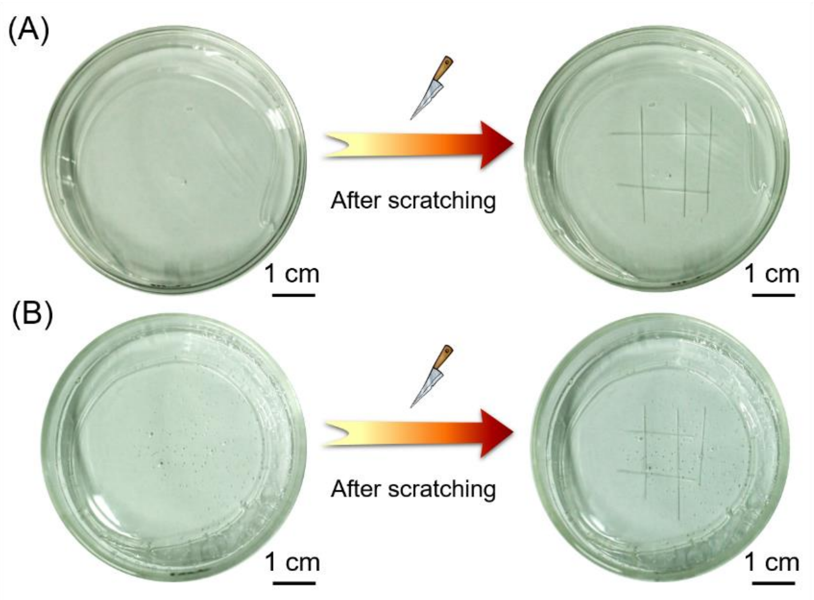

Figure S27. Photographs of PVA films containing (A) neither microcapsules nor catalyst, and (B) 5 wt% DABCO catalyst before and after being scratched with a utility knife for 10 min.

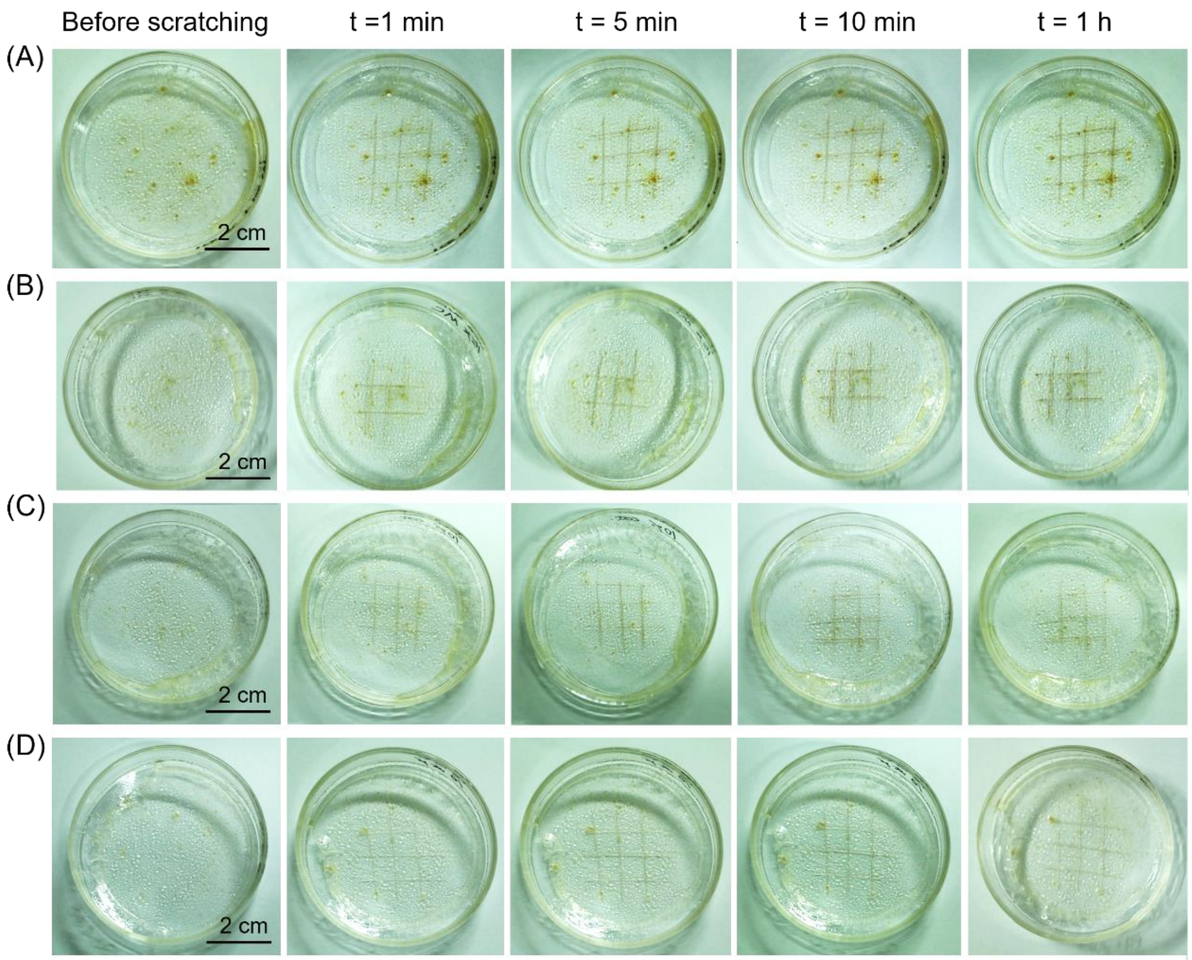

Figure S28. Photographs of PVA films containing different amount of alkyne/EPA microcapsules (alkyne content = 5.0 wt%) and 10 wt% DABCO catalyst: (A) 15 wt% alkyne/EPA microcapsules and 10 wt% DABCO catalyst, (B) 15 wt% alkyne/EPA microcapsules and 5 wt% DABCO catalyst. (C) 10 wt% alkyne/EPA and 10 wt% DABCO catalyst, and (D) 10 wt% alkyne/EPA microcapsules and 5 wt% DABCO catalyst. The photographs were taken before and after the films being scratched with a utility knife for different time. All the photographs share the same scale bar of 2 cm.

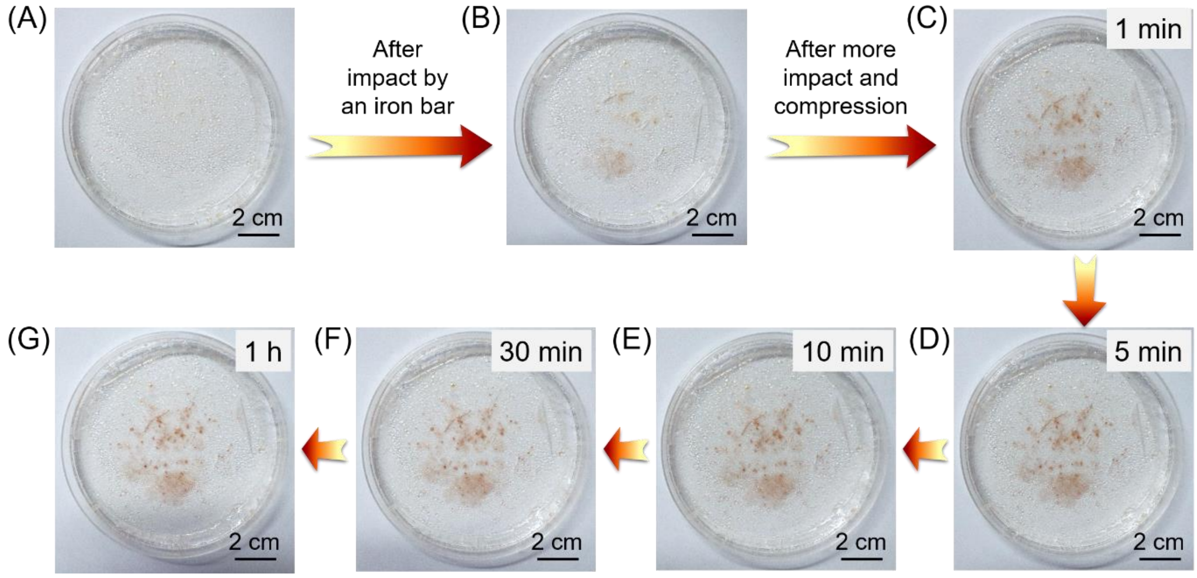

Figure S29. Photographs of PVA films containing 15 wt% alkyne/EPA microcapsules and 5 wt% DABCO catalyst before and after being impacted and compressed by an iron bar for different time.
